# Insight into the mechanism of action of temporin-SHa, a new broad-spectrum antiparasitic and antibacterial agent

**DOI:** 10.1371/journal.pone.0174024

**Published:** 2017-03-20

**Authors:** Zahid Raja, Sonia André, Feten Abbassi, Vincent Humblot, Olivier Lequin, Tahar Bouceba, Isabelle Correia, Sandra Casale, Thierry Foulon, Denis Sereno, Bruno Oury, Ali Ladram

**Affiliations:** 1 Sorbonne Universités, UPMC Univ Paris 06, CNRS, Institut de Biologie Paris-Seine (IBPS), Biogenèse des Signaux Peptidiques (BIOSIPE), Paris, France; 2 Sorbonne Universités, UPMC Univ Paris 06, CNRS, Laboratoire de Réactivité de Surface (LRS), Paris, France; 3 Sorbonne Universités, UPMC Univ Paris 06, Ecole Normale Supérieure, CNRS, Laboratoire des Biomolécules, Paris, France; 4 Department of Chemistry, Ecole Normale Supérieure, PSL Research University, UPMC Univ Paris 06, CNRS, Laboratoire des Biomolécules, Paris, France; 5 Sorbonne Universités, UPMC Univ Paris 06, CNRS, Institut de Biologie Paris-Seine (IBPS), Plate-forme Interactions Moléculaires, Paris, France; 6 Institut de Recherche pour le Développement (IRD), UMR 224 IRD-CNRS-Univ Montpellier 1 et 2 Maladies infectieuses et Vecteurs: écologie, génétique, évolution et contrôle (MiVegec), Montpellier, France; 7 IRD, UMR 177 IRD-CIRAD, Interactions Hôtes-Vecteurs-Parasites-Environnement dans les maladies tropicales négligées dues aux Trypanosomatidae (InterTryp), Montpellier, France; Nanyang Technological University, SINGAPORE

## Abstract

Antimicrobial peptides (AMPs) are promising drugs to kill resistant pathogens. In contrast to bacteria, protozoan parasites, such as *Leishmania*, were little studied. Therefore, the antiparasitic mechanism of AMPs is still unclear. In this study, we sought to get further insight into this mechanism by focusing our attention on temporin-SHa (SHa), a small broad-spectrum AMP previously shown to be active against *Leishmania infantum*. To improve activity, we designed analogs of SHa and compared the antibacterial and antiparasitic mechanisms. [K^3^]SHa emerged as a highly potent compound active against a wide range of bacteria, yeasts/fungi, and trypanosomatids (*Leishmania* and *Trypanosoma*), with leishmanicidal intramacrophagic activity and efficiency toward antibiotic-resistant strains of *S*. *aureus* and antimony-resistant *L*. *infantum*. Multipassage resistance selection demonstrated that temporins-SH, particularly [K^3^]SHa, are not prone to induce resistance in *Escherichia coli*. Analysis of the mode of action revealed that bacterial and parasite killing occur through a similar membranolytic mechanism involving rapid membrane permeabilization and depolarization. This was confirmed by high-resolution imaging (atomic force microscopy and field emission gun-scanning electron microscopy). Multiple combined techniques (nuclear magnetic resonance, surface plasmon resonance, differential scanning calorimetry) allowed us to detail peptide-membrane interactions. [K^3^]SHa was shown to interact selectively with anionic model membranes with a 4-fold higher affinity (K_D_ = 3 x 10^−8^ M) than SHa. The amphipathic α-helical peptide inserts in-plane in the hydrophobic lipid bilayer and disrupts the acyl chain packing via a detergent-like effect. Interestingly, cellular events, such as mitochondrial membrane depolarization or DNA fragmentation, were observed in *L*. *infantum* promastigotes after exposure to SHa and [K^3^]SHa at concentrations above IC_50_. Our results indicate that these temporins exert leishmanicidal activity via a primary membranolytic mechanism but can also trigger apoptotis-like death. The many assets demonstrated for [K^3^]SHa make this small analog an attractive template to develop new antibacterial/antiparasitic drugs.

## Introduction

Antimicrobial peptides (AMPs) are a ubiquitous group of natural compounds that play a major role in the innate immune system [1, 2]. Because of their ability to rapidly kill a wide range of microorganisms by inducing the lysis of their membranes and/or acting on intracellular targets [3], these peptides are less susceptible to induce microbial resistance. Naturally occurring AMPs, such as those from amphibians, are considered promising candidates for the development of therapeutic drugs, including anti-infective agents to treat resistant pathogens as well as anticancer, antidiabetic and immunomodulatory agents [4, 5].

Amphibian AMPs of the temporin family [6–10] represent particularly attractive potential therapeutic candidates. These peptides are synthesized from precursors of the dermaseptin superfamily and display the characteristic structural features: a highly conserved N-terminal region (signal peptide followed by an acidic propiece) and a hypervariable C-terminal region encoding the progenitor sequence of the AMP [11, 12]. Mature temporins share unique properties that distinguish them from other AMPs. These peptides have a small size, usually 13–14 residues [13]. However, we recently isolated an atypical member of the temporin family containing only 8 amino acid residues, named temporin-SHf (FFFLSRIF_NH2_), which is the smallest natural linear AMP found to date, with the highest percentage of Phe residues (50%) for any known peptide or protein [14]. In contrast to most AMPs, temporins have a low net positive charge (0 to +3). All temporins are C-terminally amidated and adopt an amphipathic α-helical structure in apolar media or in membrane mimetic environments [15–17]. Recently, such structure for a temporin was also observed in a media containing bacterial cells [18], using a previous circular dichroism protocol that was used for the first time to study the secondary structure of AMPs (cecropin A and magainin-2) in the presence of *E*. *coli* cells [19]. This amphipathic α-helical structure enables the temporins to interact with and destabilize microbial cytoplasmic membrane, thereby promoting membrane permeabilization and/or disruption via a “carpet-like” mechanism that can involve the formation of toroidal pores, channel aggregates or more complex structures depending on the concentration, length and sequence of the peptide [13, 15, 20, 21]. At very high peptide concentrations, the membrane can be disintegrated in a detergent-like manner.

Temporins have a relatively narrow spectrum of activity, predominantly against Gram-positive bacteria [7–9]. However, a few members of this family display a wider spectrum, with potent activity against Gram-negative bacteria and yeasts [22–25]. Moreover, a small number of temporins are able to kill parasites. Currently, only six temporins have been reported to have leishmanicidal activity (Table 1). Other amphibian AMPs with a larger size were also shown to act on *Leishmania* parasites, such as dermaseptin-S1 (34 residues), the first discovered leishmanicidal peptide, or bombinins H2 (20 residues) and H4 (21 residues) [26, 27].

**Table 1:** Temporins reported to have leishmanicidal activity.

Temporin	Sequence	Net charge^a^	Reference
Ta	FLPLIGRVLSGIL_NH2_	+2	[28]
Tb	LLPIVGNLLKSLL_NH2_	+2	[28]
SHa	FLSGIVGMLGKLF_NH2_	+2	[22]
SHd	FLPAALAGIGGILGKLF	+2	[23]
Tl	FVQWFSKFLGRIL_NH2_	+3	[29]
Tf	FLPLIGKVLSGIL_NH2_	+2	[29]

^a^ pH 7.4.

Different AMP antiparasitic activities are observed depending on the nature of the AMP and the parasite and also on the stage of the parasite. Several antiparasitic mechanisms have been described [30–32], which involve disruption of the parasite membrane in the case of temporin-Ta and Tb [28] or activity against intracellular targets in the case of histatine-5 [33]. The fact that amastigotes (the intracellular form in the vertebrate host) are generally more resistant to AMPs compared to promastigotes (the extracellular form in the insect vector) suggests that the surface composition of the *Leishmania* parasites is important and that the negatively charged glycocalyx of promastigotes, composed mainly of lipophosphoglycan (LPG) and proteophosphoglycan (PPG), plays a significant role in AMP activity. A recent study by Eggimann and collaborators indicated that PPG is a major factor for the activity of temporin-SHa against *L*. *mexicana* promastigotes and that the lack of PPG and LPG on the surface increases the resistance of this *Leishmania* species to temporins [29]. Temporins are among the smallest natural antiparasitic peptides reported to have activity against both promastigotes and amastigotes. Moreover, it appears that the small size and low charge of temporins favor diffusion across the glycocalyx into the plasma membrane. Therefore, these peptides may be useful tools to elucidate the antiparasitic mechanism and are also attractive candidates to reinforce the limited arsenal of chemotherapeutic agents for which there is evidence of emerging resistance, such as pentavalent antimonials [34–36] or miltefosine [37].

We previously showed that temporin-SHa (SHa) has a broad-spectrum activity toward Gram-positive and Gram-negative bacteria, yeasts and *Leishmania* parasites [22]. This peptide inhibited the growth of *L*. *infantum* promastigotes and axenic amastigotes. Based on the complex balance of structural and physicochemical parameters (length, secondary structure, net positive charge, hydrophobicity, helicity and amphipathicity) that govern the antimicrobial activity of AMPs [4, 38–41], we designed substituted analogs of SHa ([K^3^]temporin-SHa: [K^3^]SHa; [A^2,6,9^]temporin-SHa: [A^2,6,9^]SHa; [A^2,6,9^, K^3^]temporin-SHa: [A^2,6,9^, K^3^]SHa) to improve the antibacterial/antiparasitic activity of the parent peptide.

In this study, the structure of the peptides and their cytotoxicity against several mammalian cells were determined, and a detailed comparison of the antibacterial and antiparasitic activities of SHa and its analog [K^3^]SHa was carried out. We screened a large panel of Gram-negative and Gram-positive bacteria of clinical interest and also various trypanosomatid parasites (*Leishmania* and *Trypanosoma*), including antibiotic-resistant strains of *S*. *aureus* and antimony-resistant *L*. *infantum*. Multipassage resistance selection was performed to determine whether bacterial resistance could occur against SHa and its analogs. Biochemical and biophysical studies allowed us to compare the antiparasitic and antibacterial mechanisms of the temporins in detail. We first carried out membrane permeabilization, depolarization and time-kill assays. The temporin-induced membrane damages were then visualized by atomic force microscopy (AFM) and field emission gun-scanning electron microscopy (FEG-SEM) imaging of bacteria and parasites. We also analyzed the typical hallmarks of apoptosis in *L*. *infantum* promastigotes, such as mitochondrial depolarization and DNA fragmentation. Finally, peptide-membrane interactions were studied by surface plasmon resonance (SPR) and differential scanning calorimetry (DSC) using models of eukaryotic and prokaryotic cell membranes and also by nuclear magnetic resonance (NMR) spectroscopy using bicelles as a membrane mimetic.

## Results

### Design of SHa analogs

**Fig 1:**
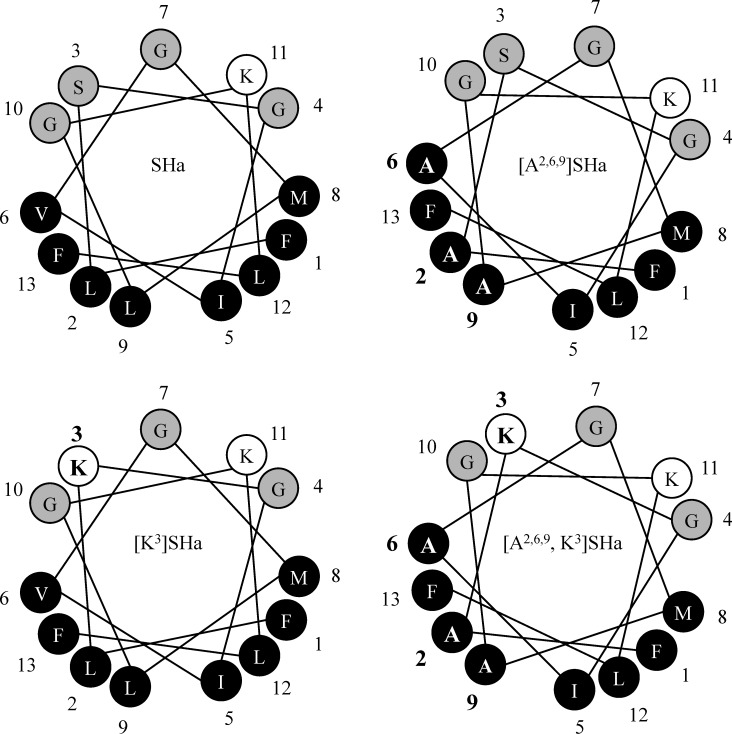
Schiffer-Edmundson helical wheel representation of temporin-SHa and its analogs. SHa, temporin-SHa; [K^3^]SHa, [K^3^]temporin-SHa; [A^2,6,9^]SHa, [A^2,6,9^]temporin-SHa; [A^2,6,9^, K^3^]SHa, [A^2,6,9^, K^3^]temporin-SHa. Apolar residues are represented in black and polar/basic residues in gray/white. Amino acid modifications are in bold. An amphipathic character, with two well-separated polar and apolar faces, is observed. Adapted from Heliquest.

The SHa analogs were designed and synthetized by modifying the structural and physicochemical parameters known to control the antimicrobial activity of AMPs, such as net positive charge, hydrophobicity, helicity and amphipathicity. Specific amino acid positions in the sequence of SHa were substituted to improve the antibacterial and antiparasitic activity of SHa while reducing its cytotoxic activity. First, the net positive charge of SHa was increased to a value of +3, yielding the analog [K^3^]SHa (Table 2). This analog has a Lys residue in position 3, which is located on the polar face of the α-helix (Fig 1), instead of a Ser residue. Because high hydrophobicity was shown to increase cytotoxic activity [4], we also designed two analogs with reduced hydrophobicity on the apolar face of the α-helix (Fig 1). The residues Leu^2,9^ and Val^6^ were replaced with Ala, yielding the analog [A^2,6,9^]SHa and also [A^2,6,9^, K^3^]SHa (Table 2). As shown by the Schiffer-Edmundson helical wheel projections (Fig 1), all analogs were predicted to conserve the amphipathic character of SHa.

**Table 2:** Sequence and physicochemical properties of temporin-SHa and its analogs.

Peptide	Sequence^a^	M_w_^b^ (Da)	Net charge^c^	<H>^d^	<*μ*H>^e^
SHa	FLSGIVGMLGKLF_NH2_	1380.76	+2	0.91	0.69
[K^3^]SHa	FL**K**GIVGMLGKLF_NH2_	1421.86	+3	0.84	0.74
[A^2,6,9^]SHa	F**A**SGI**A**GM**A**GKLF_NH2_	1268.55	+2	0.63	0.51
[A^2,6,9^, K^3^]SHa	F**AK**GI**A**GM**A**GKLF_NH2_	1309.64	+3	0.56	0.57

^a^ The substituted residues are indicated in bold in the sequence of the analogs.

^b^ Theoretical average molecular mass using Peptide Mass Calculator v3.2 (http://rna.rega.kuleuven.ac.be/masspec/pepcalc.htm).

^c^ pH 7.4.

^d^ Hydrophobicity and

^e^ hydrophobic moment were calculated using HeliQuest (http://heliquest.ipmc.cnrs.fr/).

### [K^3^]SHa displays more potent antimicrobial activity than SHa

**Fig 2:**
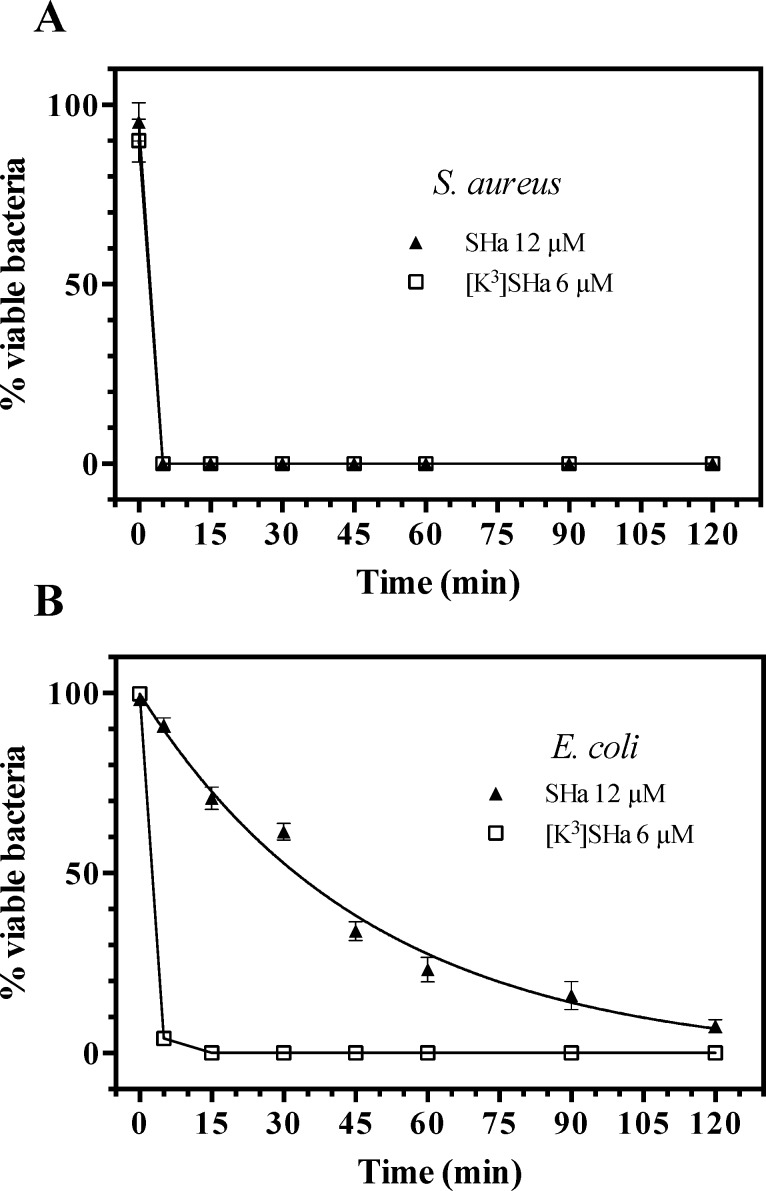
**Time-killing curves of SHa and its analog [K**^**3**^**]SHa against *S*. *aureus* ST1065 (A) and *E*. *coli* ML-35p (B).** Bacteria (10^6^ cfu/ml) were incubated in phosphate-buffered saline (PBS) with temporins at concentrations 2-fold above the MIC obtained for *S*. *aureus* ST1065 (6 *μ*M for [K^3^]SHa and 12 *μ*M for SHa). The negative control corresponds to bacteria incubated in PBS without peptide (w/o peptide). The data are shown as the means ± SEM from a single experiment carried out in triplicate and are representative of three independent experiments.

We first investigated the antimicrobial activity of SHa and analogs by determining minimal inhibitory concentrations (MICs) against a wide panel of Gram-negative and Gram-positive bacteria and yeasts/fungi (Table 3). The two analogs [A^2,6,9^]SHa and [A^2,6,9^, K^3^]SHa were inactive against all strains (MIC > 200 *μ*M). These two peptides were then used as negative controls. In contrast, the analog [K^3^]SHa was found to be highly active against all the tested bacteria (MIC = 1–6 *μ*M) and yeasts/fungi (MIC = 3–25 *μ*M), including the multidrug-resistant strains of the clinically relevant pathogenic species *S*. *aureus*, ATCC 43300 and ATCC BAA-44. A significant increase in activity was observed for this analog compared to the parent peptide, especially for Gram-negative bacteria and yeasts/fungi (Table 3). Indeed, for Gram-negative strains, MIC values decreased by approximately 8-fold for *P*. *aeruginosa*, a species that frequently develops high intrinsic resistance to many conventional antibiotics, 4-fold for *S*. *enterica* (serotype Enteritidis) and *K*. *pneumoniae*, 3-fold for *E*. *coli*, and 2-fold for *A*. *baumannii*. Moreover, [K^3^]SHa was 4-fold more potent against yeasts/fungi, even toward the SHa-insensitive species *C*. *parapsilosis* (MIC = 25 *μ*M). Table 3 also indicates that [K^3^]SHa retained the high potency of the parent peptide against *S*. *aureus* ATCC 25923 (MIC = 3 *μ*M) but was slightly more active toward other Gram-positive bacteria (2-fold) and significantly more active toward *S*. *pyogenes* (6-fold). The geometric mean of the MIC values (MIC_GM_) was calculated for several strains of the Table 3 to provide an overall evaluation of the antimicrobial activity against Gram-negative bacteria, Gram-positive bacteria and yeast/fungi, and then to use it for the determination of the therapeutic index of SHa and [K^3^]SHa. MIC_GM_ values indicated in Table 3 (in bold) underline the higher antimicrobial potency of [K^3^]SHa, particularly against Gram-negative bacteria and yeasts/fungi. At a concentration 2-fold above the MIC, temporin-SHa (12 *μ*M) and [K^3^]SHa (6 *μ*M) were both able to completely kill Gram-positive bacteria, such as *S*. *aureus*, within 5 min (Fig 2A). However, at similar concentrations, [K^3^]SHa was more efficient than SHa in killing Gram-negative bacteria (Fig 2B). While a 120-min exposure was needed for approximately 93% killing by SHa, only 15 min was needed for the [K^3^]SHa analog to achieve complete lethality in *E*. *coli* cells.

**Table 3:** Antibacterial activity of temporin-SHa analogs compared to the parent peptide.

	MIC ^a^, MIC_GM_^b^ (*μ*M)			
	SHa	[K^3^]SHa	[A^2,6,9^]SHa	[A^2,6,9^, K^3^]SHa
**Gram-negative bacteria**				
* E*. *coli* ATCC 25922	10	3	> 200	> 200
* E*. *coli* ML-35p	10	3	> 200	> 200
* P*. *aeruginosa* ATCC 27853	50	6	> 200	> 200
* S*. *enterica*^c^	25	6	> 200	> 200
* A*. *baumannii* ATCC 19606	6	3	> 200	> 200
* K*. *pneumoniae* ATCC 13883	12	3	> 200	> 200
	**14.4**	**3.8**	-	-
**Gram-positive bacteria**				
* S*. *aureus* ATCC 25923	3	3	> 200	> 200
* S*. *aureus* ST1065	6	3	> 200	> 200
* S*. *aureus* ATCC 43300^d^	6	3	-	-
* S*. *aureus* ATCC BAA-44^e^	6	3	-	-
* S*. *pyogenes* ATCC 19615	6	1	> 200	100
* L*. *ivanovii*	6	3	> 200	> 200
* E*. *faecalis* ATCC 29212	12	6	> 200	> 200
	**6**	**2.8**	-	-
**Yeasts/fungi**				
* C*. *albicans* ATCC 90028	25	6	> 200	> 200
* C*. *parapsilosis* ATCC 22019	100	25	> 200	> 200
* S*. *cerevisiae*	12	3	> 200	> 200
	**31.1**	**7.7**	-	-

^a^ Values represent the means of three independent experiments performed in triplicate.

^b^ MIC_GM_ denotes the geometric mean of the MIC values (indicated in bold) for all Gram-negative, Gram-positive and yeast/fungal strains.

^c^ Serotype Enteritidis.

^d^ Resistant to methicillin and oxacillin.

^e^ Resistant to amoxicillin/clavulanic acid, cephalothin, ciprofloxacin, erythromycin, gentamicin, imipenem, oxacillin, penicillin, tetracycline, ampicillin, doxycycline, methicillin, azithromycin, ceftriaxone, clindamycin, lincomycin, perfloxacin, rifampin, and tobramycin.

**Fig 3:**
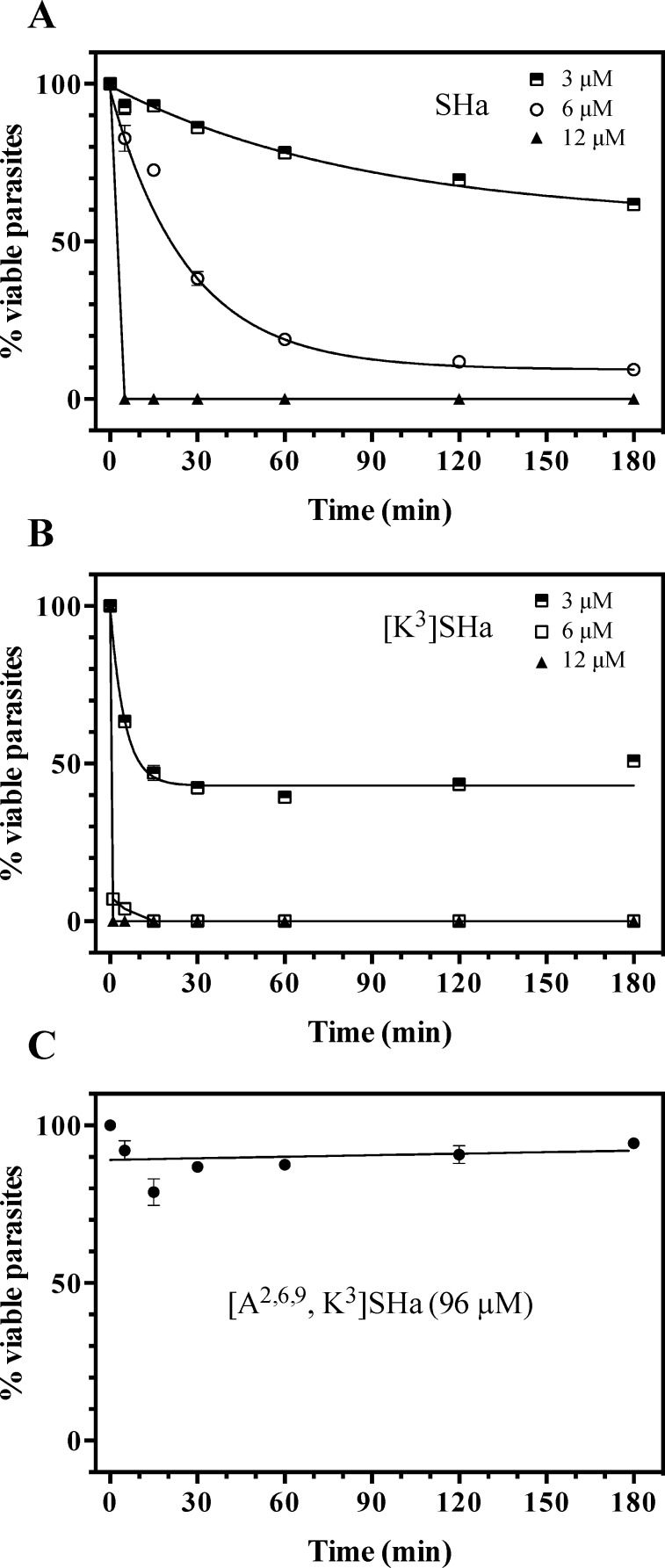
Time-kill curves of temporins against *L*. *infantum*. Parasites (2 x 10^6^ cells/ml) were incubated in HBSS with various concentrations (3, 6 and 12 *μ*M) of synthetic SHa (A) and [K^3^]SHa (B). HBSS without peptide (w/o peptide) or containing 96 *μ*M [A^2,6,9^, K^3^]SHa was used as a negative control (C). The data are shown as the means ± SEM of one representative experiment obtained from three independent experiments carried out in duplicate.

Leishmanicidal activity was analyzed on both promastigotes and amastigotes (axenic and intramacrophagic). Potent and rapid antiprotozoal activities were obtained for SHa and its analog [K^3^]SHa (Table 4 and Fig 3). The effect of temporin-SHa against *L*. *infantum* can be visualized in real time (S1 Movie). As shown in Table 4, a growth inhibitory effect was observed with promastigotes of several species of *Leishmania* responsible for visceral (*L*. *infantum*), cutaneous (*L*. *major* and *L*. *amazonensis*) and mucocutaneous (*L*. *braziliensis*) leishmaniases, with slightly higher activity for [K^3^]SHa (IC_50_ values in the range of 5–10 *μ*M) compared to the parent peptide SHa (IC_50_ = 7–18 *μ*M). We noticed a 2-fold improvement of leishmanicidal activity (IC_50_ = 8 *μ*M for SHa and 5 *μ*M for [K^3^]SHa) when peptides were incubated with *L*. *infantum* promastigotes in serum-free medium. Moreover, time-kill curves revealed clear differences in the parasite-killing efficiency of temporin-SHa (Fig 3A) and [K^3^]SHa (Fig 3B). While both peptides were active against *L*. *infantum* promastigotes at 3 *μ*M (38% and 49% killing for SHa and [K^3^]SHa, respectively, at 180 min), [K^3^]SHa induced death instantly at 12 *μ*M (within the first min) compared to 5 min for SHa. However, at 6 *μ*M, [K^3^]SHa was able to rapidly (15 min) and completely kill promastigotes, whereas a significant reduction in the number of parasites (~ 91%) was observed only after a 180-min incubation with temporin-SHa. Consistent with their lack of antibacterial effect, [A^2,6,9^]SHa and [A^2,6,9^, K^3^]SHa showed no antiparasitic activity against *L*. *infantum* promastigotes (IC_50_ > 200 *μ*M, Table 4). As shown in Fig 3C, no killing was observed when parasites were incubated with 96 *μ*M [A^2,6,9^, K^3^]SHa. As observed for antibiotic-susceptible and antibiotic-resistant *S*. *aureus* strains, [K^3^]SHa and SHa retained the ability to kill *L*. *infantum* parasites susceptible and resistant to antimony with the same efficiency (Table 4). Interestingly, leishmanicidal activity was demonstrated for [K^3^]SHa (IC_50_ = 5 *μ*M) and SHa (IC_50_ = 9 *μ*M) against intramacrophagic amastigotes as well as against the axenic forms (IC_50_ = 20 *μ*M) (Table 4). Other trypanosomatids, such as *T*. *brucei gambiense* (responsible for sleeping sickness) and *T*. *cruzi* (the etiological agent of Chagas disease), were susceptible to temporins (Table 4). The activity against *T*. *brucei gambiense* was quite similar for SHa and [K^3^]SHa (IC_50_ ~ 15 *μ*M) but *T*. *cruzi* was slightly more susceptible to the peptide analog (~ 2-fold). The trypanocidal effect of temporin-SHa is shown in the S2 Movie.

**Table 4:** Antiprotozoal activity of temporin-SHa and [K^3^]temporin-SHa.

	IC_50_ (*μ*M)	
	SHa	[K^3^]SHa
***Leishmania* promastigotes**		
* L*. *infantum*^a^	18 (8)^b^	10 (5)^b^
* L*. *infantum* (antimony resistant)	14	9
* L*. *major*	13	10
* L*. *braziliensis*	7	5
* L*. *amazonensis*	13	8
***Leishmania* amastigotes**		
* L*. *infantum* (axenic)	20	20
* L*. *infantum* (intramacrophagic)	9	5
***Trypanosoma* epimastigotes**		
* T*. *brucei gambiense*	14	16
* T*. *cruzi*	17	10

^a^ [A^2,6,9^]SHa and [A^2,6,9^, K^3^]SHa were inactive against *L*. *infantum* promastigotes (IC_50_ > 200 *μ*M).

^b^ values in parentheses were obtained in serum-free medium.

As a positive control, we also evaluated the leishmanicidal effect of dermaseptin B2, a potent 33-residue AMP isolated from frog skin (*Phyllomedusa bicolor*) that kills microorganisms by the carpet mechanism [42]. A high leishmanicidal activity against *L*. *infantum* promastigotes was observed for this peptide (IC_50_ = 2.5 *μ*M).

### [K^3^]SHa displays a better therapeutic index

The cytotoxicity of temporins was evaluated toward rat and human erythrocytes, and several human cell lines (Table 5). At antimicrobial concentrations (3–6 *μ*M for bacteria and virtually all yeast strains; 5–15 *μ*M for parasites), low cytotoxicity (human erythrocytes, THP-1 monocytes and THP-1-derived macrophages) or no cytotoxicity (HepG2 cells and fibroblasts) was observed for [K^3^]SHa. For SHa, similar results were obtained, with a 2-fold higher hemolytic activity on human erythrocytes (25 *μ*M) compared to [K^3^]SHa. However, this 2-fold higher activity was also found for both peptides on rat erythrocytes. These results indicate that intramacrophagic amastigotes can be killed by temporins without damage to the host cell at leishmanicidal concentrations. As for bacteria and parasites, the analogs [A^2,6,9^]SHa and [A^2,6,9^, K^3^]SHa were inactive toward erythrocytes, and no cytotoxicity has been detected when [A^2,6,9^]SHa was also tested on THP-1 monocytes (IC_50_ > 60 *μ*M) and HepG2 cells (IC_50_ > 600 *μ*M) (Table 5). In contrast, the positive control, dermaseptin B2, was highly cytotoxic toward THP-1 monocytes (IC_50_ ~ 7 *μ*M).

**Table 5:** Cytotoxic activity of temporins SHa and analogs against human cells and rat erythrocytes.

	IC_50_ or LC_50_ (*μ*M)			
Cells	SHa	[K^3^]SHa	[A^2,6,9^]SHa	[A^2,6,9^, K^3^]SHa
Rat erythrocytes	25	26	> 200	> 200
Human erythrocytes	25	50	> 100	ND
Human THP-1 monocytes	> 60	48	> 60	ND
Human THP-1-derived macrophages	60	55	ND	ND
Human HepG2	560	358	> 600	ND
Human fibroblasts	> 100	> 100	ND	ND

Values were determined with GraphPad Prism 6.0 software and are the means of three independent experiments performed in triplicate. IC_50_, half maximal inhibitory concentration; LC_50_, half maximal lytic concentration (erythrocytes and macrophages).

ND: not determined.

To evaluate the cell selectivity (microorganisms versus human cells), the therapeutic indices of [K^3^]SHa and SHa were calculated as the ratio of IC_50_ or LC_50_ values for the different human cells over the MIC_GM_ values obtained for Gram-negative bacteria, Gram-positive bacteria and yeasts/fungi. The results showed in Table 6 reveal that [K^3^]SHa displays a better therapeutic index with values 1.37-fold to 8.12-fold higher than those of SHa depending on the type of microorganisms and cells considered. The therapeutic indices of [K^3^]SHa were also better when they were calculated with LC_50_ values against rat erythrocytes (indicated in Table 5) and MIC_GM_: Gram-negative bacteria, T.I.(SHa) = 1.7, T.I.([K^3^]SHa) = 6.8 (4-fold higher); Gram-positive bacteria, T.I.(SHa) = 4.2, T.I.([K^3^]SHa) = 9.3 (2.21-fold higher); yeasts/fungi, T.I.(SHa) = 0.8, T.I.([K^3^]SHa) = 3.4 (4.25-fold higher). Therefore, the overall results indicate greater antimicrobial specificity for the analog [K^3^]SHa compared to the parent peptide.

**Table 6:** Therapeutic indices (T.I.) of [K^3^]SHa and SHa^a^.

T.I.^b^	Human	THP1-	THP1-derived	HepG2	Fibroblasts
[K^3^]SHa (SHa)	erythrocytes	monocytes	macrophages		
Gram-negative strains	13.1 (1.7)	12.6 (8.3)	14.5 (4.2)	94.2 (38.9)	52.6 (13.9)
	**7.70**^**c**^	**1.52**	**3.45**	**2.42**	**3.78**
Gram-positive strains	17.8 (4.2)	17.1 (20)	19.6 (10)	127.8 (93.3)	71.4 (33.3)
	**4.24**	**0.85**	**1.96**	**1.37**	**2.14**
Yeast/fungal strains	6.5 (0.8)	6.2 (3.8)	7.1 (1.9)	46.5 (18)	26 (6.4)
	**8.12**	**1.63**	**3.37**	**2.58**	**4.06**

^a^ Therapeutic indices of SHa are indicated in parentheses.

^b^ Ratio of IC_50_ or LC_50_ values (for human cells from Table 5) over the geometric mean of MIC values (from Table 3 and corresponding to Gram-negative, Gram-positive, and yeast/fungal strains). When IC_50_ or LC_50_ values were higher than the maximum concentration tested, a minimal 2-fold concentration value was used to calculate the therapeutic index because IC_50_ or LC_50_ values were determined by carrying out serial 2-fold dilutions (for example, LC_50_ > 100 was considered as 200).

^c^ Values in bold represent the fold improvement in the therapeutic index of [K^3^]SHa compared to SHa.

### [K^3^]SHa alters more efficiently the integrity of the bacterial and parasite plasma membrane

**Fig 4:**
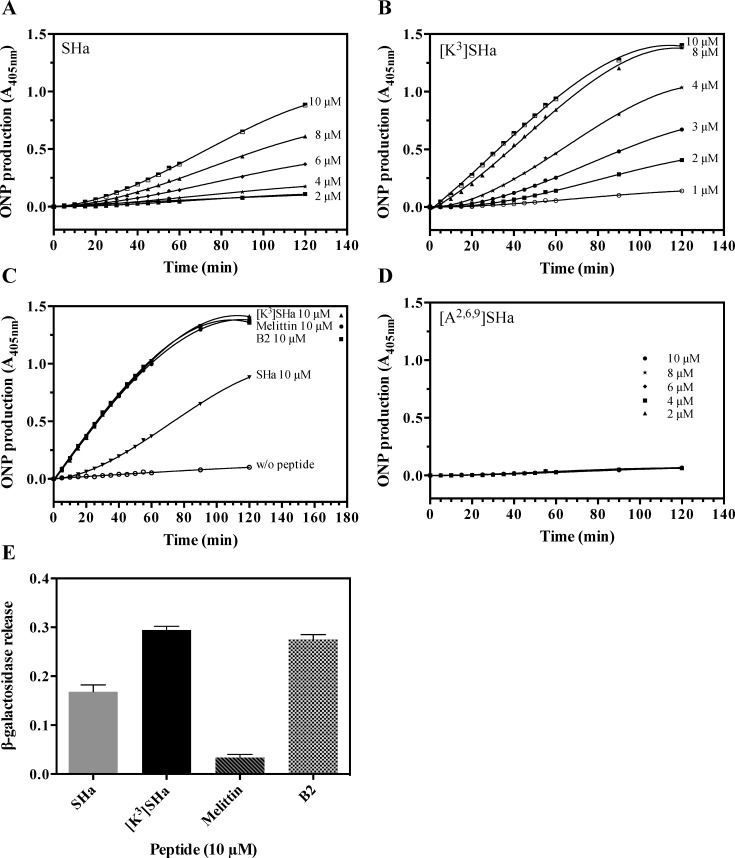
Temporin-induced membrane permeabilization of *E*. *coli* ML-35p. Bacteria were incubated with different concentrations of SHa (A) or [K^3^]SHa (B). The leakage kinetics were measured as the production of ONP at 405 nm resulting from hydrolysis of ONPG by the cytoplasmic bacterial β-galactosidase. C, comparison of the membrane leakage of temporins (SHa and [K^3^]SHa), dermaseptin B2 (B2) and melittin at the same concentration (10 *μ*M). The negative control without peptide is also indicated (w/o peptide). D, no permeabilization was observed with [A^2,6,9^]SHa (2, 4, 6, 8 and 10 *μ*M). E, Extracellular release of cytoplasmic β-galactosidase after 60 min incubation of bacteria with 10 *μ*M peptide followed by centrifugation (1,000 x g, 10 min, 4°C) to measure ONP production (A_405_) in the supernatant. The results are expressed as the means ± SEM after subtraction of the negative control values (no peptide) from the test values and correspond to one representative experiment carried out in triplicate.

The effects of temporins on the membrane integrity of bacteria and parasites were investigated by two complementary approaches (ONPG and SYTOX Green assays) to analyze membrane permeabilization. First, we incubated SHa and [K^3^]SHa with *E*. *coli* ML-35p, a bacterial strain constitutively expressing cytoplasmic β-galactosidase, and we measured the time-dependent hydrolysis of the small chromogenic substrate *o*-nitrophenyl-β-D-galactopyranoside (ONPG) into *o*-nitrophenol (ONP) by cytoplasmic β-galactosidase (Fig 4). As indicated in Fig 4A and 4B, both peptides were able to permeabilize the bacterial cytoplasmic membrane. However, [K^3^]SHa induced a more rapid and potent permeabilization of the Gram-negative strain *E*. *coli* ML-35p compared to the parent peptide. Interestingly, at 10 *μ*M, this 13-residue analog was as efficient as the two positive controls used in our study, dermaseptin B2 (33 residues) and melittin (26 residues), with identical kinetics of permeabilization (Fig 4C). Under the same conditions, the level of permeabilization of SHa was 1.6-fold lower than that of [K^3^]SHa. In contrast, the analog [A^2,6,9^]SHa was not able to permeabilize the cytoplasmic membrane of *E*. *coli* ML-35p (Fig 4D). This is consistent with its lack of antimicrobial activity. To assess the extent of membrane damage caused by temporins and to determine whether these peptides act via pore formation or a detergent-like effect, we investigated the release of intracellular β-galactosidase (Mw ~ 540 kDa, Stokes radius ~ 70 Å) from *E*. *coli* ML-35p bacteria upon incubation with 10 *μ*M peptide. As shown in Fig 4E, the leakage induced by [K^3^]SHa led to extracellular β-galactosidase activity that was comparable to dermaseptin B2 but 29-fold higher than melittin (a pore-forming peptide) [43]. This leakage was 1.7-fold higher than that induced by SHa. Thus, these results suggest that temporins and dermaseptin B2 act through similar mechanisms, i.e., a detergent-like effect [42], and also confirm the potent bactericidal activity of [K^3^]SHa by inducing a more efficient disruption of the bacterial membrane.

**Fig 5:**
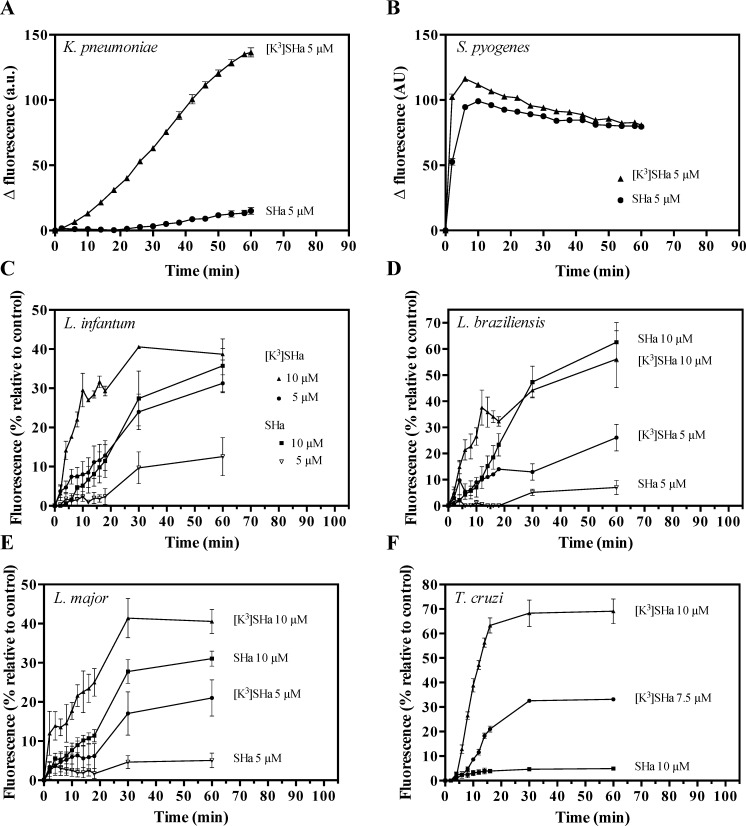
**Temporin-induced SYTOX Green (SG) influx into the bacteria *K*. *pneumoniae* ATCC 13883 (A) and *S*. *pyogenes* ATCC 19615 (B), and the parasites *L*. *infantum* (C), *L*. *braziliensis* (D), *L*. *major* (E), and *T*. *cruzi* (F).** Bacteria (10^6^ cfu/ml) and parasites (2.5 x 10^6^ cells/ml) were preincubated with 1 *μ*M SG, and peptides (SHa or [K^3^]SHa) were added after fluorescence stabilization. Membrane alteration is correlated with the fluorescence of the DNA fluorescent probe (λ_excitation_ = 485 nm and λ_emission_ = 520 nm). For bacteria, the data are expressed as the means ± SEM after subtraction of the negative control values (w/o peptide) from the test values. For parasites, the results (mean ± SEM) were plotted as a percentage of the fluorescence relative to that of parasites fully permeabilized by 0.1% Triton X-100. The curves correspond to one experiment carried out in triplicate and are representative of two independent experiments.

In the second approach to analyze membrane permeabilization, we used two clinically relevant strains (*S*. *pyogenes* and *K*. *pneumoniae*), and we tested the ability of the vital dye SYTOX Green (SG, ~ 600 Da), which requires lesions in the membrane, to enter into the cell. For the Gram-positive *S*. *pyogenes*, SHa and [K^3^]SHa showed rapid and similar SG influx kinetics at 5 *μ*M (Fig 5A). However, for the Gram-negative strain *K*. *pneumoniae*, the [K^3^]SHa analog more efficiently permeabilized the cell membrane compared to SHa (Fig 5B). These results are consistent with the ONPG experiments and confirm the high antibacterial efficiency of the analog [K^3^]SHa against Gram-negative and Gram-positive strains. To elucidate the antiparasitic mechanism of temporins, we analyzed the permeabilization of the *Leishmania* and *Trypanosoma* cytoplasmic membranes. As shown in Fig 5C–5F, parasite permeabilization occurred in a distinct curve-shaped manner, with a more rapid and potent SG influx into *Leishmania* and *T*. *cruzi* parasites for [K^3^]SHa compared to the parent peptide. Differences were clearly observed after 60 min incubations with the two peptides, particularly at 5 *μ*M, where 12% (*L*. *infantum*), 7% (*L*. *braziliensis*) and 5% (*L*. *major*) of the maximum permeabilization were reached after incubation with SHa *vs* 31% (*L*. *infantum*), 26% (*L*. *braziliensis*) and 21% (*L*. *major*) with [K^3^]SHa. Moreover, the influx of SG into *T*. *cruzi* was 14-fold higher after incubation of the parasites with 10 *μ*M [K^3^]SHa (69% of permeabilization at 60 min, only 5% for SHa) (Fig 5F). This was consistent with the enhanced antitrypanosomal activity of the analog (Table 4).

**Fig 6:**
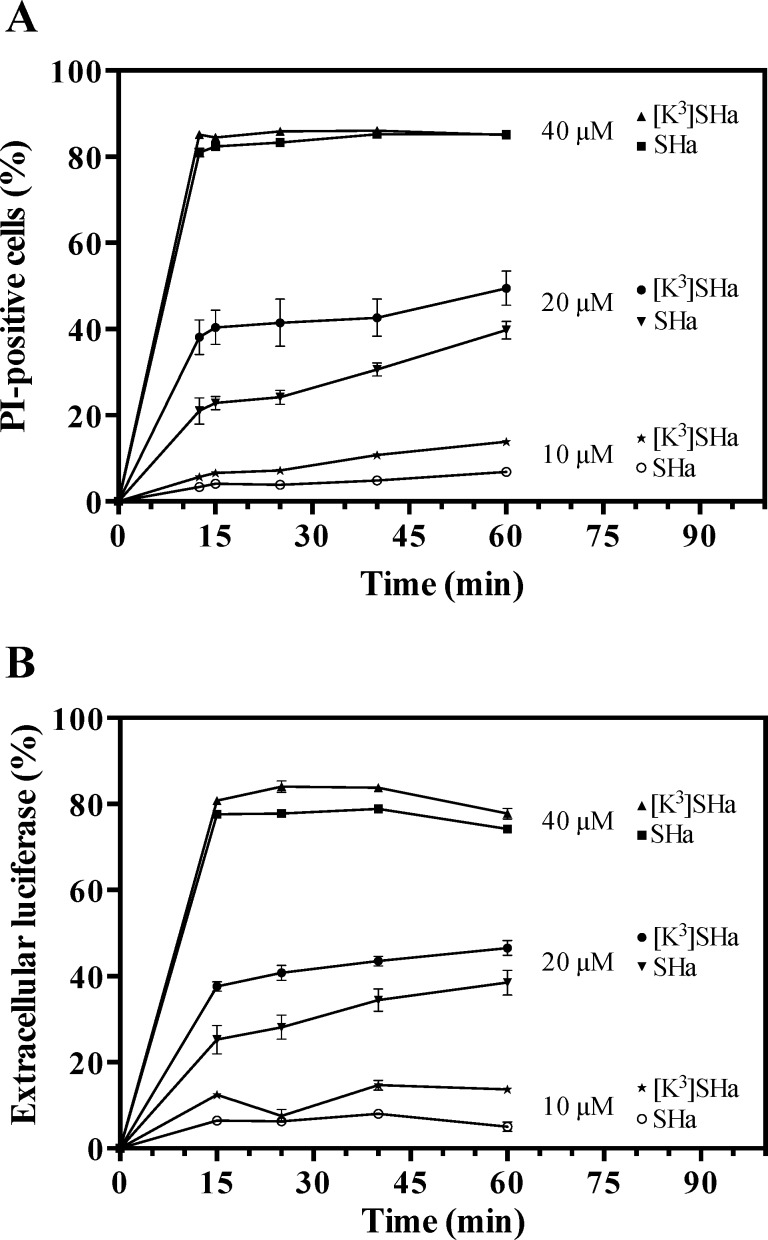
**Dose- and time-dependent propidium iodide (PI) staining (A) and luciferase release in the extracellular medium (B) of *L*. *infantum* parasites upon addition of temporins.** *L*. *infantum* promastigotes (10^6^ cells/ml) were incubated with different concentrations (10, 20 and 40 *μ*M) of SHa or [K^3^]SHa for different times. PI-positive cells were counted by flow cytometry after adding PI (1 *μ*g/ml) to the parasites. The luciferase activity in the extracellular medium was determined after centrifugation of the parasites and measurement of the luminescence using the Steady-Glo® Luciferase Assay System (Promega). The data are expressed as the means ± SEM of two experiments carried out in triplicate.

When we used propidium iodide (PI), another nucleic acid stain, [K^3^]SHa was also more efficient in affecting the integrity of the *L*. *infantum* membrane at 10 and 20 *μ*M (Fig 6A). At 40 *μ*M, similar PI staining curves were obtained for both temporins (85% of permeabilized cells). Using the same methodology for bacteria, we investigated the damage to *L*. *infantum* promastigotes caused by temporins by evaluating the leakage of intracellular components, such as luciferase (~ 61 kDa). Peptide-induced leakage of luciferase was detected by measuring enzyme activity in the supernatant at various times (Fig 6B). The curves were similar to those obtained with PI staining.

**Fig 7:**
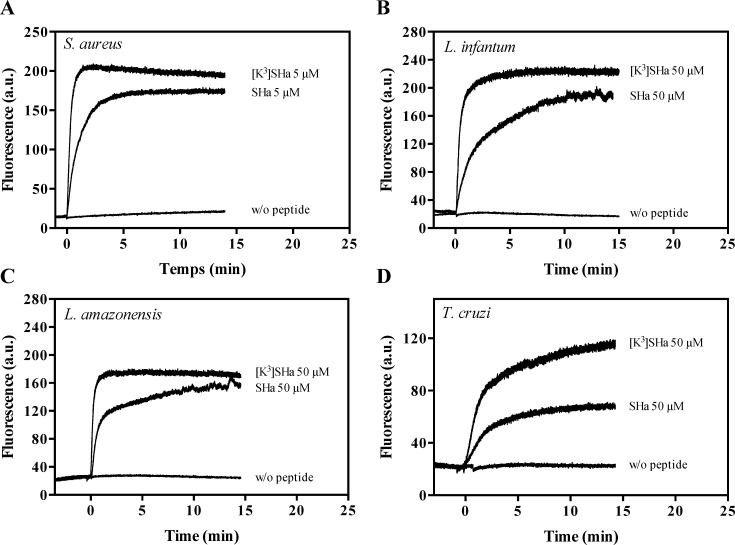
Changes in the membrane potential of bacteria and parasites upon addition of temporins. *S*. *aureus* ATCC 25923 (A), *L*. *infantum* (B), *L*. *amazonensis* (C) and *T*. *cruzi* (D) were equilibrated with DiSC_3_(5) (1 *μ*M for *S*. *aureus* and 2.5 *μ*M for parasites). SHa or [K^3^]SHa was then added (t = 0) at a concentration of 5 *μ*M (bacteria) or 50 *μ*M (parasites), and changes in the fluorescence were monitored for 15 min (bacteria) or 20 min (parasites) at λ_excitation_ = 622 nm and λ_emission_ = 670 nm. The curves correspond to a single experiment representative of three independent experiments.

Temporins were tested for their ability to dissipate the membrane potential using the fluorescent cyanine dye DiSC_3_(5) (3,3′-dipropylthiadicarbocyanine iodide). We observed that the addition of the peptides caused an instantaneous depolarization of the *S*. *aureus* and *L*. *infantum* membranes, resulting in increased fluorescence (Fig 7A and 7B). This effect was more potent for [K^3^]SHa, as indicated by the fact that a plateau with an increased magnitude was reached within 1.5 min (*S*. *aureus*) or 2.5 min (*L*. *infantum*). Membrane depolarization was also detected for *L*. *amazonensis* and *T*. *cruzi*, although to a lesser extent for the latter (Fig 7C and 7D).

Altogether, the killing and permeabilization/depolarization experiments are consistent with membrane impairment and the release of cellular components, two concomitant events of the primary mode of action of temporins that kill bacteria and parasites.

**Fig 8:**
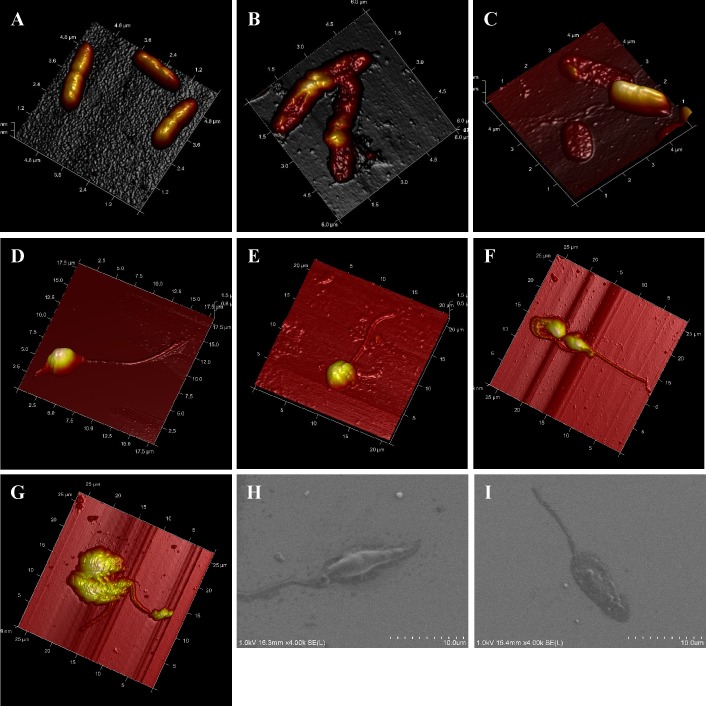
**AFM and FEG-SEM visualization of the effect of temporins-SH on *P*. *aeruginosa* bacteria (A–C) and parasites (*L*. *infantum* promastigotes and *T*. *cruzi* epimastigotes; D–I).** A–C, AFM imaging of *P*. *aeruginosa*: A, untreated control bacteria; B, bacteria after 1 h incubation with 50 *μ*M SHa; C, bacteria treated for 1 h with 6 *μ*M [K^3^]SHa. Bacteria were severely damaged by temporins compared to the control. D–G, AFM imaging of *L*. *infantum* promastigotes and *T*. *cruzi* epimastigotes: D and E, *L*. *infantum* untreated (D) or treated with 5 *μ*M [K^3^]SHa (E); F and G, *T*. *cruzi* without peptide (F) or with 5 *μ*M [K^3^]SHa (G). H and I, FEG-SEM visualization of *L*. *infantum* promastigotes without peptide (H) or with 5 *μ*M [K^3^]SHa. Morphological changes were observed for parasites that were incubated with peptides (E, G and I).

We used atomic force microscopy (AFM) and field emission gun-scanning electron microscopy (FEG-SEM) imaging to obtain high-resolution images of the effects of temporins on bacterial and parasite morphology. As shown in Fig 8A and 8B, when the pathogenic Gram-negative bacteria *P*. *aeruginosa* was in contact with 50 *μ*M SHa for 1 h, severe membrane damage with drastic morphological changes of the bacteria were observed (Fig 8B) compared to the untreated control bacteria (Fig 8A). Fig 8C indicates that *P*. *aeruginosa* cells were also severely damaged by [K^3^]SHa at a very low concentration (6 *μ*M), as revealed by the presence of flattened bacteria. In this selected area of the glass surface, bacteria with a normal shape were also present. This is likely due to the low peptide concentration (6 *μ*M, corresponding to the MIC) relative to the high cell concentration (4 x 10^7^ cells/ml, i.e., a bacterial density 40-fold higher than that used for MIC determination). Incubation of *L*. *infantum* promastigotes or *T*. *cruzi* epimastigotes with 5 *μ*M [K^3^]SHa for 30 min led to loss of the morphological integrity of the parasites (Fig 8D–8G). AFM imaging revealed temporin-induced damage of the cell body and the flagellum, with alterations of the parasite shape (Fig 8E and 8G) compared to untreated parasites (Fig 8D and 8F). FEG-SEM analysis of *L*. *infantum* showed that 5 *μ*M [K^3^]SHa caused permeabilization/lysis of the parasite membrane, likely with leakage of the cellular contents, leading to a large, flattened leaf-shaped parasite (Fig 8H and 8I). Thus, microscopic analysis indicated that the primary killing effect of temporins is similar for bacteria and trypanosomatids and occurs through a membranolytic mechanism.

### SHa and its analog induce apoptotic-like death in *Leishmania infantum* promastigotes

**Fig 9:**
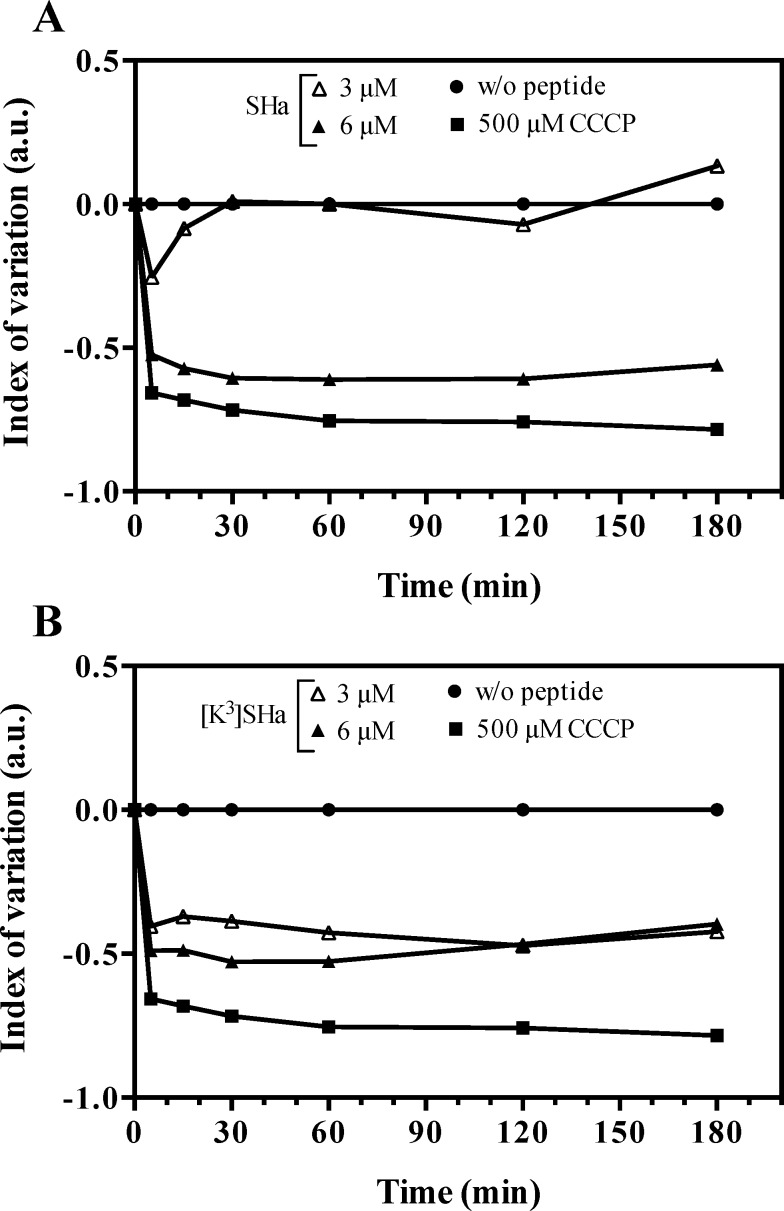
Kinetics of mitochondrial membrane depolarization of *L*. *infantum* promastigotes. Parasites were incubated for 3 h at 26°C with different concentrations of peptide (3 and 6 *μ*M, final concentrations). Mitochondrial membrane potential was monitored by flow cytometry using the fluorescence probe TMRE. A, index of variation for SHa. B, index of variation for [K^3^]SHa. Negative and positive controls were assayed without peptides or with 500 *μ*M CCCP, respectively. The index of variation is expressed in arbitrary units (a.u.). The curves were obtained from a single experiment representative of three independent experiments.

Because temporins permeabilize the parasite membrane even at doses below lytic concentrations, we investigated whether these peptides could induce cell death by apoptosis. As a first step, we used the fluorescence probe tetramethylrhodamine ethyl ester (TMRE) to monitor the mitochondrial membrane potential in *L*. *infantum* promastigotes (Fig 9). As indicated by the negative index of variation (IV) values, SHa (Fig 9A) and [K^3^]SHa (Fig 9B) were able to depolarize the mitochondrial membrane compared to the positive (CCCP: carbonyl cyanide m-chlorophenylhydrazone, a well-known uncoupling agent) and negative (no peptide) controls. However, while both peptides showed similar depolarization profiles at 6 *μ*M, the mitochondrial membrane potential was not affected at a 2-fold lower concentration of SHa, unlike [K^3^]SHa.

**Fig 10:**
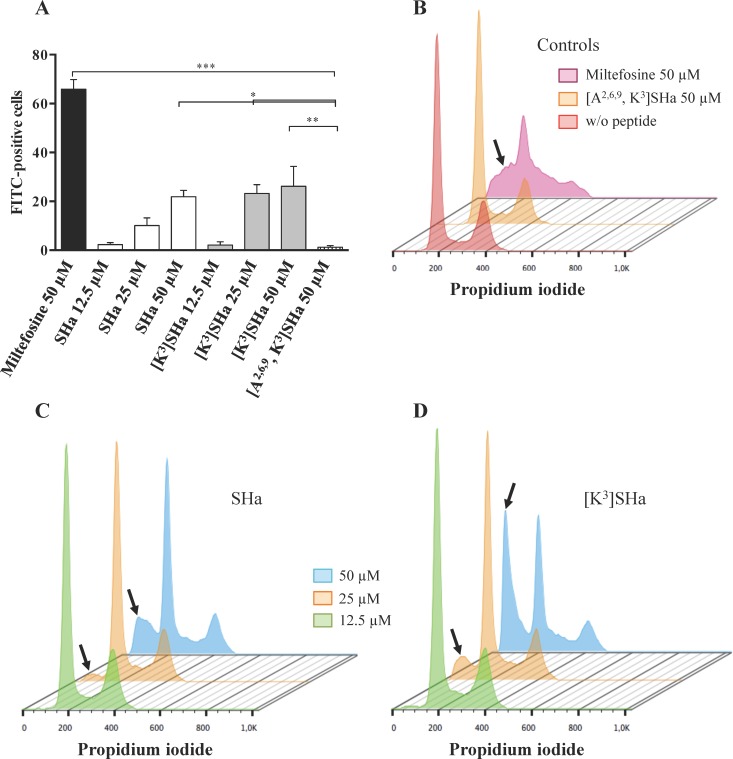
**DNA fragmentation (A) and cell cycle analysis (B–D) of *L*. *infantum* promastigotes.** Parasites were treated with different concentrations (12.5, 25 and 50 *μ*M, final concentrations) of SHa or [K^3^]SHa. Miltefosine (hexadecylphosphocholine, 50 *μ*M), a drug used for the treatment of leishmaniasis that is known to induce apoptosis, and [A^2,6,9^, K^3^]SHa (50 *μ*M) were used as positive and negative controls, respectively. A, DNA fragmentation was assessed by TUNEL assay, and fluorescence values were corrected (subtraction of negative control fluorescence value) and converted into a histogram that represents the percentage of FITC-positive cells. Parametric data were analyzed by a one-way ANOVA and Dunnett’s post-test using GraphPad Prism 5.0. *, p < 0.05; **, p < 0.01; ***, p < 0.001. B-D, *L*. *infantum* promastigotes were stained with propidium iodide and analyzed by flow cytometry. Flow cytograms are shown: B, parasites untreated or treated with 50 *μ*M of [A^2,6,9^, K^3^]SHa or miltefosine; C, parasites treated with SHa (12.5, 25 *μ*M or 50 *μ*M); D, parasites treated with [K^3^]SHa (12.5, 25 *μ*M or 50 *μ*M). The sub-G1 peak is shown with an arrow. Flow cytograms correspond to a single experiment representative of three independent experiments and were obtained using FlowJo vX.0.7 software.

Next, we analyzed DNA fragmentation, another hallmark of apoptosis. For this assay, parasites were incubated with antileishmanial compounds for 48 h, and DNA fragmentation was monitored by TUNEL (terminal deoxynucleotidyl transferase-mediated dUTP nick-end labeling) assay (Fig 10A) or by cell cycle analysis (Fig 10B–10D). As shown in Fig 10A, when *L*. *infantum* promastigotes were treated with 50 *μ*M miltefosine (hexadecylphosphocholine), a drug used for leishmaniasis treatment that is known to induce apoptosis [44], approximately 65% of DNA fragmentation was observed. In contrast, at the same concentration, [A^2,6,9^, K^3^]SHa did not cause any fragmentation. In the presence of SHa, a dose-dependent effect was observed, with a significant DNA fragmentation (approximately 25%) at 50 *μ*M. A similar level of DNA fragmentation was observed when parasites were treated with a 2-fold lower concentration of [K^3^]SHa (25 *μ*M) and was maintained at 50 *μ*M (Fig 10A). These results were confirmed by cell cycle analysis of *L*. *infantum* promastigotes (Fig 10B–10D). Indeed, flow cytograms revealed a sub-G1 peak after treatment with SHa (50 *μ*M) or [K^3^]SHa (25 and 50 *μ*M) (Fig 10C and 10D) compared to the negative controls (Fig 10B), indicating a significant sub-G1 population (Table 7). At a concentration of 50 *μ*M, similar percentages of sub-G1 cells were observed for miltefosine (29%) and SHa (26%). For [K^3^]SHa, the proportion of sub-G1 cells was concentration-dependent, with percentages of 18% and 47% at 25 and 50 *μ*M, respectively (Table 7). At concentrations above the IC_50_, significant apoptosis-like death of *L*. *infantum* promastigotes was observed following incubation with SHa and [K^3^]SHa.

**Table 7:** Percentage of *L*. *infantum* promastigotes in the sub-G1 phase of the cell cycle.

Compound	Sub-G1 phase (% of cells)
**Control**	
* *No peptide	4.9
* *[A^2,6,9^, K^3^]SHa 50 *μ*M	0.85
* *Miltefosine 50 *μ*M	29.1
**SHa**	
* *12.5 *μ*M	5.68
* *25 *μ*M	8.81
* *50 *μ*M	26.5
**[K**^**3**^**]SHa**	
* *12.5 *μ*M	6.61
* *25 *μ*M	18.3
* *50 *μ*M	46.9

### Multipassage resistance selection reveals that temporins display no propensity to promote bacterial resistance, unlike ampicillin

**Fig 11:**
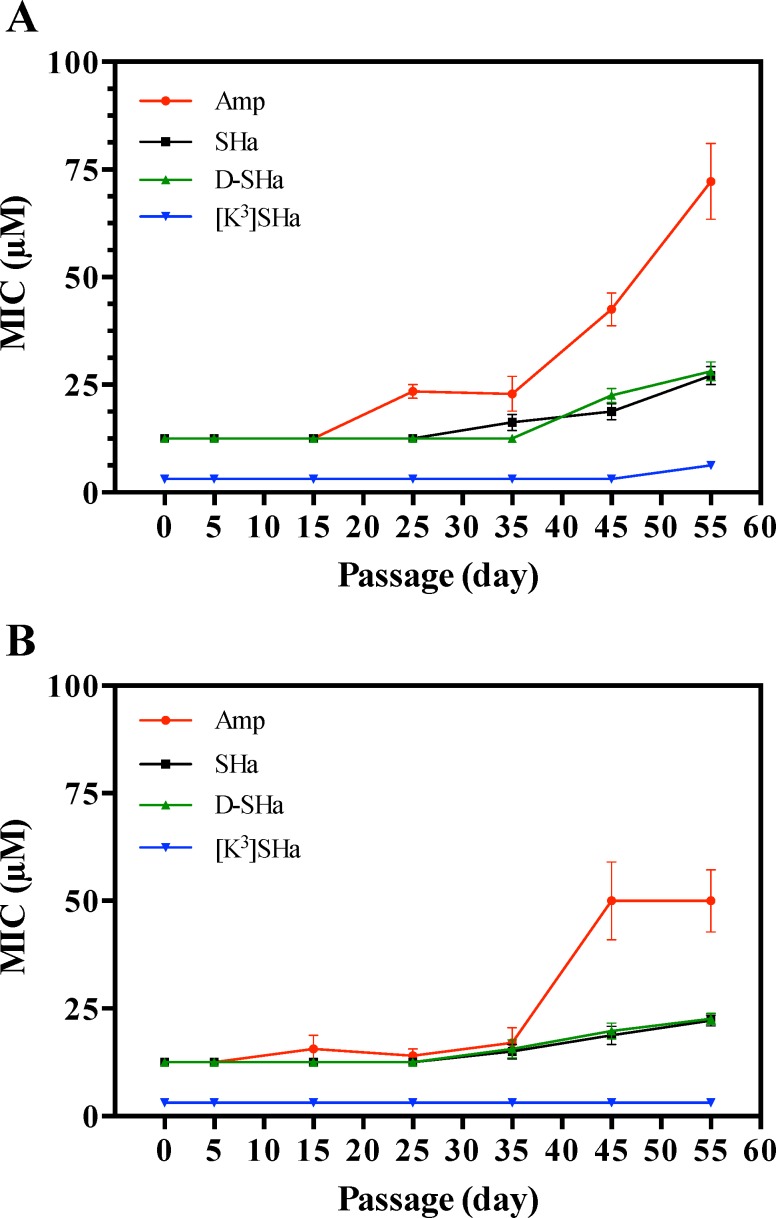
Multipassage resistance selection. A, plot of MICs against *E*. *coli* lineages adapted to increasing concentrations of temporins or ampicillin. B, control: MICs against lineages grown in the same conditions without antimicrobial agents (MilliQ water). The following temporins were tested: SHa, D-SHa (SHa with all residues in D-configuration), and [K^3^]SHa. The conventional antibiotic ampicillin was also used for comparison. *E*. *coli* ATCC 25922 was continuously re-cultured in the presence of doubling concentrations of antimicrobial agents from 1/16 of the MIC until adaptation to the MIC (50 passages, 10 bacterial lineages with 1/16 MIC, 1/8 MIC, 1/4 MIC, 1/2 MIC, and MIC) (see Materials and Methods). The MIC of the antimicrobial agent was determined against the adapted *E*. *coli* lineages originating from different last passages: passage 5 (*E*. *coli* with no antimicrobial agent), 15 (*E*. *coli* with a concentration of antimicrobial agent equal to 1/16 MIC), 25 (1/8 MIC), 35 (1/4 MIC), 45(1/2 MIC), and 55 (MIC). MIC values were obtained in triplicate and represent the average of three independent experiments. Curves representing the MIC as a function of the passage number were obtained from the means ± SEM of MIC values of at least three independent experiments.

To determine whether bacterial resistance may emerge against temporins, we performed experiments on long-term cultures to select resistant mutants using SHa and [K^3^]SHa. The more stable analog D-SHa (enantiomeric SHa) with all residues in the D-configuration was also tested because it is potentially less prone to induce bacterial resistance. We used *E*. *coli* ATCC 25922, and we selected ampicillin as a conventional antibiotic for comparison. The MIC values of temporins and ampicillin were as follows: SHa, 12.5 *μ*M; [K^3^]SHa, 3 *μ*M; D-SHa, 12.5 *μ*M; ampicillin, 12.5 *μ*M. In our resistance selection assay (see Materials and Methods), *E*. *coli* ATCC 25922 was exposed to increasing concentrations of temporins or ampicillin from MIC/16 to MIC (50 passages, 10 bacterial lineages with MIC/16, MIC/8, MIC/4, MIC/2 and MIC) after 5 initial passages in unsupplemented Mueller-Hinton (MH) medium. A control with MilliQ H_2_O instead of an antimicrobial agent was also performed using the same conditions. After selection, the MICs of temporins and ampicillin were determined against the adapted lineages originating from different last passages (passage 5, *E*. *coli* with no antimicrobial agent; 15, *E*. *coli* with a concentration of antimicrobial agent equal to MIC/16; 25, MIC/8; 35, MIC/4; 45, MIC/2; 55, MIC) (Fig 11A), and also against control bacteria (H_2_O) corresponding to the same passages that were not subjected to antibacterial agent adaptation (Fig 11B). During the 55 passages, we observed a constant MIC value for [K^3^]SHa (3 *μ*M) against control bacteria (Fig 11B). SHa and D-SHa displayed similar profiles, with an approximate 2-fold increase in the MIC value at the end of the selection. However, unlike temporins, bacteria became naturally less susceptible to ampicillin. Indeed, an increase in the MIC value occurred from day 35 to reach a 4-fold greater value until day 55 (Fig 11B). Interestingly, we observed no more than a 2-fold increase in the MIC when temporins were tested against adapted *E*. *coli* lineages (Fig 11A). Because resistance is defined as a > 4-fold increase in MIC [45, 46], this indicates that temporin resistance did not develop during the 55 passages in our conditions. This was not the case for ampicillin, where a 6-fold increase over the initial MIC was detected at the end of the selection (Fig 11A).

### [K^3^]SHa forms an amphipathic α-helix oriented parallel to the lipid membrane and binds selectively to anionic lipids with a higher affinity than SHa

**Fig 12:**
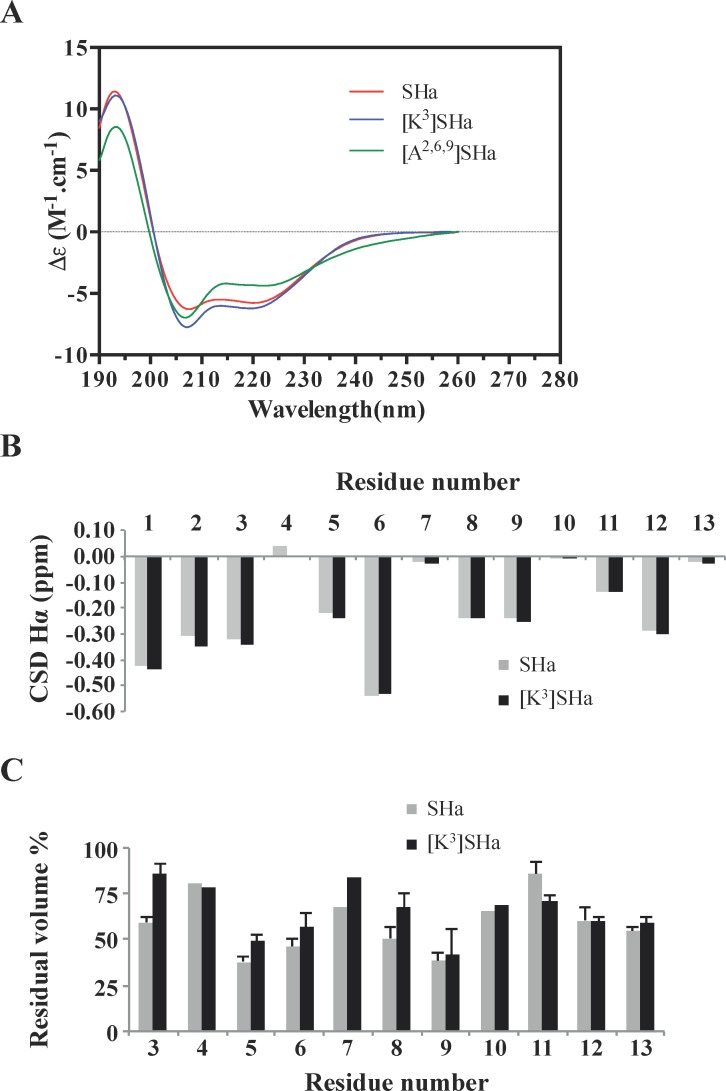
CD and NMR investigation of temporins. A, CD spectra of SHa, [K^3^]SHa and [A^2,6,9^]SHa (30 *μ*M) in DMPC:DMPG 3:1 (mol:mol) LUVs (1 mg/ml in PBS). No ordered structure was found in PBS. CD measurements are reported as the dichroic increment (Δε) per residue. The relative helix content was deduced as the percent of helix = [Δε_222_ x –10], where Δε_222 nm_ is the dichroic increment at 222 nm. B, NMR chemical shift deviations (CSDs) of Hα protons of SHa and [K^3^]SHa in 50 mM DHPC/25 mM DMPG bicelles. C, Residual peak volume after addition of 2% 1-palmitoyl-2-stearoyl-(12-doxyl)-*sn*-glycero-3-phosphocholine (12-doxylPC) paramagnetic probe. For each residue, 1 to 3 cross-peaks corresponding to HN-Hα and HN-Hβ NOE correlations were integrated. The HN protons of residues 1 and 2 were not detected. The standard deviation of peak volumes integrated for each residue is indicated.

We previously demonstrated by circular dichroism (CD) and nuclear magnetic resonance (NMR) spectroscopy that SHa adopts an α-helical conformation in water/2,2,2-trifluoroethanol mixtures and in sodium dodecyl sulfate detergent micelles [15]. The secondary structure of SHa and its analogs was investigated by CD in the presence of dimyristoyl phosphatidylcholine (DMPC) / dimyristoyl phosphatidylglycerol (DMPG) 3:1 (mol:mol) anionic large unilamellar vesicles (LUVs) as a bacterial membrane mimic (Fig 12A). SHa, [K^3^]SHa and [A^2,6,9^]SHa adopt α-helical ordered structures, as evidenced by the characteristic double minima at 208 and 222 nm, with global helical contents of 57%, 61% and 44%, respectively. The conformations of the two membrane-active peptides were next studied by NMR. We chose phospholipid bicelles made of short-chain zwitterionic DHPC (dihexanoyl phosphatidylcholine) and long-chain anionic DMPG as a more reliable membrane mimic than micelles [47]. Indeed, bicelles are disc-shaped assemblies that have an advantage over detergent micelles as they can form a planar phospholipid bilayer. A long-chain to short-chain phospholipid *q* ratio of 0.5 was chosen to form small, isotropically tumbling bicelles in solution. The ^1^H resonance linewidths of temporins in bicelles were larger than in detergent micelles, as expected for the higher molecular weight peptide-bicelle complex, but they were still compatible with high-resolution solution NMR studies. The ^1^H chemical shifts of SHa and the [K^3^]SHa analog (S1 and S2 Tables) were assigned using 2D TOCSY and 2D NOESY experiments optimized for the selective detection of amide/aromatic protons in the acquisition dimension, as the non-deuterated lipids displayed strong signals in the aliphatic region. The conformation of both peptides was analyzed at the residue level using the chemical shift deviation (CSD) of Hα protons (Fig 12B). CSDs are defined as the difference between experimental and random coil chemical shifts and are good indicators of secondary structure formation [48]. Both peptides had large negative values of Hα CSDs that are characteristic of helical conformation. The [K^3^]SHa analog displayed slightly more negative CSD values in the first turn of the helix, indicating that the Ser to Lys substitution marginally stabilized the helix structure, consistent with the CD results. Both peptides also exhibited a large number of HN-HN and Hα-HN nuclear Overhauser effects (NOEs) that are characteristic of α-helical conformations, namely strong intraresidual Hα_i_-HN_i_ and sequential HN_i_-HN_i+1_ NOE correlations, sequential Hα_i_-HN_i+1_ and medium-range HN_i_-HN_i+2_, Hα_i_-HN_i+3_ and Hα_i_-HN_i+4_ NOEs. The sequence of both temporins contains 3 Gly, residues known to have a weak helical propensity. Although the CSDs of the Gly Hα protons dropped to zero, helical-type NOEs were still observed on either side of the Gly residues, suggesting that the helical structures are not interrupted. The use of non-deuterated lipids enabled us to detect intermolecular NOEs between peptides and phospholipids. The aromatic side chain protons of Phe^1^ and Phe^13^ form NOEs with the protons at 1.25 ppm, corresponding to the methylenic groups (C4 to C12) of lipid acyl chains, indicating that the temporins penetrate through their hydrophobic face into the hydrocarbon bilayer. Phe^1^, but not Phe^13^, also form NOEs with the glycerol backbone protons of phospholipids, indicating that it adopts orientations that are more exposed in the membrane than Phe^13^. We also used a lipophilic paramagnetic probe to more precisely analyze the orientation of temporins relative to the bilayer. The addition of 2% 1-palmitoyl-2-stearoyl-(12-doxyl)-*sn*-glycero-3-phosphocholine (12-doxylPC) in bicelles led to differential broadening of the proton resonances. The most affected resonance corresponds to the Phe^13^ Hζ proton in both peptides (10% residual intensity), indicating a close proximity to the paramagnetic probe. The paramagnetic relaxation enhancements were monitored at the residue level by measuring residual volumes of the HN-Hα and HN-Hβ correlations on 2D NOESY spectra (Fig 12C). Periodic variations of the residual amplitudes could be detected: residues Ile^5^, Val^6^ and Leu^9^ on the hydrophobic face were the most affected, while residues Gly^4^, Gly^7^, Gly^10^ and Lys^11^ on the polar face were the least affected. These paramagnetic waves clearly indicate that both SHa and [K^3^]SHa adopt parallel orientations with respect to the bilayer surface. The Ser to Lys substitution induced differences in paramagnetic relaxation enhancements. Lys^3^ was less affected and therefore more exposed than Ser^3^ in the lipid bilayer. It is likely that a closer positioning to the surface is induced by favored electrostatic interactions between the Lys side chain and anionic lipid headgroup.

To obtain further insight into the mechanism of interaction by which [K^3^]SHa exerts its membranolytic effect, we used differential scanning calorimetry (DSC). Changes in the thermodynamics of lipid interactions and lipid phase transitions were monitored to assess the ability of the peptide to interact with and disrupt the lipid acyl chain packing compared to the parent peptide SHa. For this study, we used negatively charged DMPG multilamellar vesicles (MLVs) as a model for bacterial membranes and zwitterionic DMPC MLVs as a model for mammalian cell membranes. As shown in S1 Fig, [K^3^]SHa, similar to SHa, abolished the pretransition of the DMPG (S1A Fig) and DMPC (S1B Fig) MLVs, starting at the lowest concentration (peptide/lipid ratio = 1:100). The weakly energetic pretransition peak of DMPG and DMPC occurs near 13°C due to the conversion of the Lβ′ (ordered lamellar gel phase with tilted hydrocarbon chains) to the Pβ′ phase (ordered rippled gel phase) [49]. The disappearance of the transition peak suggests that electrostatic interactions occur between the peptide and the lipid headgroups, resulting in the abolition of hydrocarbon chain tilt in the gel phase bilayer. Very different perturbing effects were detected on the main lipid-phase transition of negatively charged and zwitterionic MLVs, a strongly energetic and highly cooperative transition with a peak appearing near 23°C (DMPG) or 24°C (DMPC) (conversion of the rippled gel phase to the fluid lamellar liquid-crystalline phase Lα) [49] (S1A and S1B Fig). The main phase transition (chain melting) is essentially due to *trans*-*gauche* isomerization of the acyl chains (i.e., a decrease in the acyl chain packing of lipid molecules), which increases the fluidity of the membrane. We observed that binding of a low concentration of [K^3^]SHa (peptide/lipid ratio = 1:100) to negatively charged MLVs led to a marked decrease in the melting temperature (T_m_) and enthalpy (ΔH) of the main phase transition, together with an enhanced broadening of the peak (ΔT_½_) (S1A Fig). Because the change in the value of ΔH upon peptide addition reflects the disruption of van der Waals interactions between the hydrocarbon chains, this shows that [K^3^]SHa is able to intercalate between the fatty acid chains, reducing the cooperativity of the transition (increase in the ΔT_1/2_). Moreover, the decrease in the melting temperature (T_m_) of the main transition indicates stabilization of the fluid lamellar liquid-crystalline phase by hydrophobic interactions between the peptide and lipid acyl chains [50–52]. Interestingly, an increased peptide amount (peptide/lipid ratio = 1:50) led to a complete abolishment of the main phase transition, indicating that [K^3^]SHa, compared to SHa, severely perturbs anionic bilayer membranes by interacting with the polar headgroups and acyl region of the phospholipids, disrupting the acyl chain packing of the bilayer. In contrast, [K^3^]SHa and SHa only slightly affected the main transition of DMPC (S1B Fig). This indicates that these peptides interact “atmospherically” at the headgroup level without penetrating and perturbing the hydrophobic core of the zwitterionic vesicles, consistent with the low cytotoxic activity of the peptide. Altogether, the DSC results demonstrate that [K^3^]SHa, similar to SHa, selectively interacts with anionic membranes. However, compared to SHa, [K^3^]SHa induces stronger perturbations in the lipid chain packing that are consistent with deep penetration of the hydrophobic region of the peptide helix into the fatty acyl chains of the lipid bilayer.

**Fig 13:**
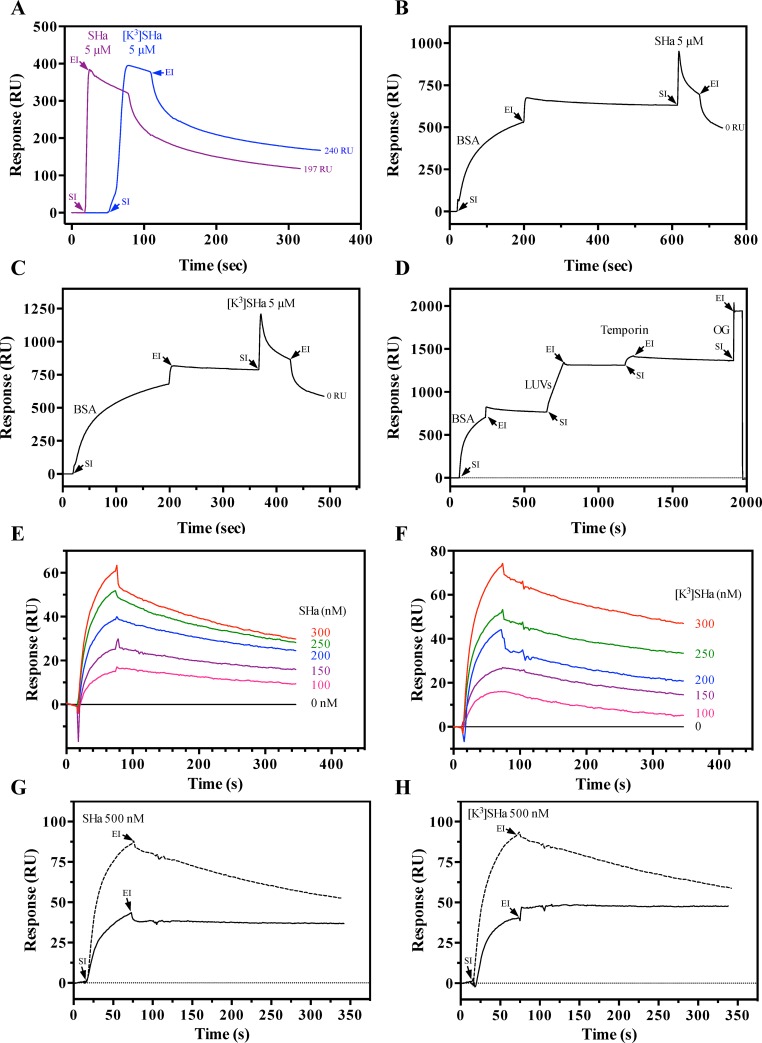
Surface plasmon resonance (SPR) analysis of temporin binding to negatively charged DMPC/DMPG 3:1 (mol:mol) LUVs. A, binding of temporins directly to the L1 sensor chip surface. SHa and [K^3^]SHa injected at a concentration of 5 *μ*M (20 *μ*l during 1 min) interact with the carboxymethylated dextran containing covalently attached alkyl chains, as indicated by the significant amount of temporin non-specific binding (SHa: 197 RU, [K^3^]SHa: 240 RU) remaining on the sensor chip surface after the end of peptide injection. B and C, binding of SHa (B) and [K^3^]SHa (C) after injection of BSA. In contrast, no peptide interaction was observed after binding of 0.2 mg/ml BSA (15 *μ*l injected during 3 min) to the sensor chip surface followed by injection of SHa or [K^3^]SHa (5 *μ*M). D, complete SPR cycle used for the binding of temporins. In the example, 0.2 mg/ml BSA was first injected (15 *μ*l during 3 min) on the L1 surface to prevent non-specific binding of temporins and was followed by an injection (2 *μ*l during 2 min) of 0.2 mg/ml DMPC/DMPG LUVs and then of peptide (300 nM of SHa in the example; 20 *μ*l during 1 min). Complete regeneration of the surface was obtained using 40 mM of the detergent *n*-octyl-β-D-glucopyranoside (OG) (30 *μ*l injected during 1 min). E and F, determination of the binding affinity of temporins SHa (E) and [K^3^]SHa (F). Peptides diluted in HBS-N buffer were tested at different concentrations (0 to 300 nM) for their binding to DMPC/DMPG LUVs. The baseline corresponds to HBS-N alone. The following K_D_ values were calculated by BIAevaluation software analysis: K_D_ (SHa) = 1.3 ± 0.4 x 10^−7^ M, χ^2^ = 3.7 ± 1.3 (n = 3); K_D_ ([K^3^]SHa) = 3.1 ± 0.7 x 10^−8^ M, χ^2^ = 3.2 ± 0.7 (n = 3). Chi^2^ (χ^2^) values below 10 indicate a good fit of the Langmuir (1:1) binding model. G and H, selective SPR binding of temporins SHa (G) and [K^3^]SHa (H) toward anionic model membranes. Negatively charged DMPG or zwitterionic DMPC LUVs were injected onto the L1 sensor chip precoated with BSA (0.2 mg/ml). Temporins (500 nM) were then injected, and binding to the DMPG (solid line) and DMPC (dashed line) LUVs was monitored. RU: resonance units; SI: start of injection; EI: end of injection. The curves correspond to a single experiment representative of three different experiments.

The membrane-binding affinity of temporins was determined by surface plasmon resonance (SPR) using negatively charged LUVs (DMPC/DMPG 3:1). When [K^3^]SHa or SHa (5 *μ*M) was injected directly onto a L1sensor chip, we observed non-specific binding of the peptide (SHa: 197 RU, [K^3^]SHa: 240 RU) to the carboxymethylated dextran containing covalently attached alkyl chains (Fig 13A). Immobilization of bovine serum albumin (BSA, 0.2 mg/ml) on the sensor chip surface prior to temporin injection led to complete abolition of this non-specific binding (Fig 13B and 13C). We therefore followed an optimized SPR procedure, shown in Fig 13D, to determine the temporin-binding equilibrium dissociation constant K_D_. Interaction kinetics were obtained using a range of temporin concentrations (Fig 13E and 13F). To optimize the calculation of the kinetic constants, the response was limited below 100 RU (resonance units) for the peptide (0–300 nM range). SHa (Fig 13E) and [K^3^]SHa (Fig 13F) both displayed high binding affinity (K_D_ range: 10^−7^ to 10^−8^ M) toward the negatively charged bacteria membrane mimetic model. However, the affinity of the analog [K^3^]SHa was approximately 4-fold higher (K_D_ = 3.1 ± 0.7 x 10^−8^ M; n = 3) than the natural peptide (K_D_ = 1.3 ± 0.4 x 10^−7^ M; n = 3). Chi-square (χ^2^) values were lower than 10 (SHa: χ^2^ = 3.7 ± 1.3; [K^3^]SHa: χ^2^ = 3.2 ± 0.7), indicating the reliable quality of the fit (1:1 Langmuir binding model). The better affinity of [K^3^]SHa is in agreement with its more potent antibacterial activity and its higher net positive charge, which may enhance electrostatic interactions with the anionic membrane. The selective interaction of temporins with anionic membranes (DMPG LUVs) revealed by DSC experiments was confirmed by SPR. When 500 nM of SHa (Fig 13G) or [K^3^]SHa (Fig 13H) was injected on DMPG LUVs immobilized on the L1 sensor chip precoated with BSA, the binding plateau of both temporins remained during the dissociation phase (end of injection). In contrast, this phenomenon was not observed with DMPC LUVs, indicating a selective and strong interaction of temporins with the negatively charged DMPG vesicles and a probable peptide insertion into the membrane.

## Discussion

The increasing prevalence of microbial drug resistance is a major public health concern that threatens the effective prevention and treatment of infections caused by various microorganisms. According to the WHO [53], antibiotic resistance has reached alarming levels in many countries of the world, with few, if any, treatment options in some cases. This is particularly true for multidrug-resistant Gram-negative bacteria that produce extended-spectrum β-lactamases (ESBLs), such as *E*. *coli*, *K*. *pneumoniae*, *P*. *aeruginosa* and *A*. *baumannii*. Drug resistance is also found in parasites. This is the case for *Leishmania*, the causative agent of leishmaniases, a group of neglected tropical diseases endemic in 98 countries, with an estimated 1.3 million new cases and 20,000–50,000 deaths reported every year [54, 55]. In most developing countries, the main treatment for leishmaniasis is antimonials (first-line drugs) [56, 57]. However, in addition to the toxic effects and the need for parenteral administration, this therapy leads to the emergence of resistance [35].

Due to their small size and simple composition, temporins are attractive compounds for the development of a new class of peptide-based anti-infective drugs. Among temporins-SH isolated from the North African ranid frog *Pelophylax saharicus*, SHa has emerged as a potent AMP, with broad-spectrum activity against Gram-positive and Gram-negative bacteria (MICs in the range of 3–50 *μ*M), yeasts/fungi, and also against *Leishmania infantum* [15, 22]. Increasing the net positive charge of SHa yielded a highly potent analog, [K^3^]SHa. This analog is more efficient than the parent peptide against a wide range of clinically relevant bacterial species, with MICs in the range 1–6 *μ*M against all bacteria and yeasts/fungi tested, except for *C*. *parapsilosis* (MIC = 25 *μ*M). The higher antimicrobial specificity of [K^3^]SHa was attested by its higher therapeutic index than SHa, which was determined from IC_50_ or LC_50_ values obtained for various human cells and from the geometric mean of MIC values obtained against several strains of Gram-negative bacteria, Gram-positive bacteria, and yeast/fungi. Substitution of Ser^3^ with Lys in the sequence of SHa retains the α-helical structure and enhances the electrostatic interactions with the membrane and consequently improves antimicrobial activity. The Schiffer-Edmundson helical wheel projections presented in Fig 1 show that the helix is amphipathic, with a narrow polar face containing Lys and Gly residues and a wide apolar face consisting of bulky hydrophobic residues (Leu, Phe). The high number of Gly residues does not appear to be detrimental to helix stability. This is likely linked to the exclusive distribution of Gly over the hydrophilic face, as is also observed for Gly-rich plasticin antimicrobial peptides [58], with helix stabilization promoted by favorable interactions of bulky side chains on the hydrophobic face.

The [K^3^]SHa helix is more amphipathic (<*μ*H> = 0.74) and less hydrophobic (<H> = 0.84) than SHa (<*μ*H> = 0.69, <H> = 0.91), which may explain the 2-fold decrease in its hemolytic activity. This is consistent with previous studies indicating the importance of the net positive charge and hydrophobicity for antimicrobial and hemolytic activity, respectively [59–62]. The difference of hemolytic activity of [K^3^]SHa observed between rat and human erythrocytes might be directly related to the wide variation in the phosphatidylcholine (PC) and sphyngomyelin (SM) content of the erythrocyte membrane in different mammalian species. Indeed, the different hemolytic susceptibility of erythrocytes from various mammalian species to amphipathic cationic peptides was related to the differences in the PC/SM ratio and then to the membrane fluidity [63]. The analogs [A^2,6,9^]SHa and [A^2,6,9^, K^3^]SHa were designed to decrease the hydrophobicity and consequently the hemolytic character. Nevertheless, an alanine insertion on the apolar face of the parent peptide led to a complete loss of antimicrobial activity as well as hemolytic/cytotoxic activity. This phenomenon was also observed for temporin-Ta [64]. Structural changes may explain the inactivity of the two alanine-substituted analogs against prokaryotes because we observed a lower α-helical content for [A^2,6,9^]SHa upon interaction with anionic model membranes by CD. This effect can be ascribed to the large reduction in side chain hydrophobicity and confirms that the helical folding of temporins is predominantly driven by hydrophobic side chain interactions with lipid acyl chains.

Time-kill studies revealed that both SHa and [K^3^]SHa were able to kill *S*. *aureus* bacteria within 5 min at a concentration 2-fold above the MIC. However, the killing effect of [K^3^]SHa against the Gram-negative bacteria *E*. *coli* was more efficient (15 min). Regarding the mechanism of action, both peptides were shown to induce a rapid permeabilization/depolarization, which was accompanied by an extracellular release of macromolecular complexes (such as β-galactosidase, ~540 kDa). At 10 *μ*M, the β-galactosidase release induced by [K^3^]SHa was equivalent to that induced by dermaseptin B2, a 33-residue AMP acting via a detergent-like mechanism. SHa (10 *μ*M) was less effective (60% of β-galactosidase leakage), and a minimal effect (only 12%) was observed with melittin (10 *μ*M), a pore-forming peptide (toroidal pore-forming mechanism) [65]. These results suggest that SHa and its analog induce bacterial death by a detergent-like effect, as has been described for temporins Ta, Tb, SHf and SHd [14, 23, 66]. To provide further insight into the mechanism, peptide-membrane interactions were analyzed by biophysical techniques using zwitterionic and anionic model membranes. SPR studies showed that both temporins were able to selectively bind anionic membranes with high affinity. However, the affinity of [K^3^]SHa was 4-fold higher (K_D_ = 3.1 ± 0.7 x 10^−8^ M) than that of the parent peptide (K_D_ = 1.3 ± 0.4 x 10^−7^ M), consistent with its improved antimicrobial activity. The enhanced interaction of [K^3^]SHa with anionic membranes was confirmed by DSC. At a peptide-lipid ratio of 1:100 or 1:50, the analog completely abolished the main lipid-phase transition of DMPG MLVs, while this phenomenon was not observed with SHa. Therefore, similar to SHf [14], [K^3^]SHa strongly and selectively perturbs anionic bilayer membranes. The in-plane insertion, as evidenced by NMR, enables the amphipathic peptide to interact with the polar headgroups and the acyl chains of the phospholipids, disrupting the acyl chain packing of the bilayer.

The small size, low cationic charge and rapid membranolytic mechanism of temporins are major assets in limiting the development of antimicrobial resistance. In our conditions, continuous exposure of *E*. *coli* to increasing concentrations of temporins did not induce bacterial resistance. We noticed only a non-significant 2-fold increase in the MIC value at the end of 55 passages, reflecting a naturally occurring phenomenon that was also observed when the peptides were tested against non-adapted *E*. *coli* lineages (negative controls). In contrast, ampicillin-adapted *E*. *coli* became resistant to the antibiotic, with a 6-fold increase in the MIC value at the end of the selection. To our knowledge, this is the first study on bacterial resistance development performed with temporins. Very few studies have been performed with small AMPs. For example, resistance to magainin-2 developed in *S*. *aureus* under consistent *in vitro* exposure to the peptide, which was due to an increase in the net charge and membrane rigidity of the bacterial strain [67]. Similarly, *Escherichia coli* and *Pseudomonas fluorescens* became resistant to pexiganan, a magainin-2 analog, after continued *in vitro* selection [68]. This development of resistance is not limited to AMPs but has also been observed with peptidomimetics, such as α-peptide/0078-peptoid [69].

Compared to bacteria, yeasts and fungi, protozoan parasites, such as *Leishmania*, have been little studied as targets of AMPs [30, 31, 70], and very few antiparasitic peptides have been identified (only 3% are listed in the APD3, Antimicrobial Peptide Database, http://aps.unmc.edu/AP/main.php). One of the main reasons is the existence of two parasitic stages, i.e., an extracellular promastigote stage (in the insect vector) and an intracellular amastigote stage (in the vertebrate host) that is more difficult to target by AMPs. Therefore, the antiparasitic mechanism of AMPs has not been extensively studied and remains unknown. We previously showed that SHa is active against *Leishmania infantum* prosmastigotes and also axenic amastigotes [22]. In this study, we evaluated the effect of this peptide and its analog [K^3^]SHa on a broader range of parasite species of the Trypanosomatidae family, including of the genera *Leishmania* (*L*. *infantum*, *L*. *major*, *L*. *braziliensis*, *L*. *amazonensis*) and *Trypanosoma* (*T*. *brucei gambiense*, *T*. *cruzi*). Both temporins were efficient (IC_50_ values in the range of 5–20 *μ*M) against all species tested and also against antimony-resistant parasites. However, differences in temporin sensitivity were observed depending on the parasitic form. Promastigotes were slightly more sensitive to the analog [K^3^]SHa, with IC_50_ values ranging from 5 to 10 *μ*M (SHa: IC_50_ = 7–18 *μ*M), whereas axenic *L*. *infantum* amastigotes were 2-fold less sensitive to [K^3^]SHa (IC_50_ = 20 *μ*M) than promastigotes. This is consistent with previous experiments indicating the importance of the glycocalyx, a surface coat present in promastigotes that contains negatively charged LPG and PPG, in the electrostatic interactions with temporins [29]. Another interesting finding concerns the intramacrophagic activity, which was higher for both temporins (IC_50_ = 5–9 *μ*M) compared to the extracellular forms (promastigotes and axenic amastigotes). These observations suggest that temporins may either directly target *Leishmania* amastigotes in the phagolysosome to kill them or act in synergy with other intracellular messengers, such as NO, which are known to be involved in the killing of *Leishmania* amastigotes [71]. Regarding the first hypothesis, we demonstrated in this study that SHa and its analog are able to enter into *Leishmania* promastigotes and reach the mitochondria to induce apoptosis. So, we cannot exclude that, according to a similar process (membrane translocation or pinocytosis), temporins could passively enter into the macrophage and target *Leishmania* amastigotes in the parasitophorous vacuole. This specific targeting of the parasitophorous vacuole membrane by the positively charged temporins could be due to the presence of negatively charged LPG that were transferred during the promastigote-induced phagosome remodeling [72]. After peptide translocation into the parasitophorous vacuole, this would enable direct killing of amastigotes. Nevertheless, other intramacrophagic deleterious events involving signaling pathways, like NO synthesis, are possible and remain to be explored. Moreover, intramacrophagic leishmanicidal activity occurred without cytotoxicity toward the host cell. This property is of particular interest in view of potential therapeutic applications of temporins as antiparasitic agents. We also observed a 2-fold higher leishmanicidal activity of temporins in serum-free medium. The effect of serum on antimicrobial activity has been previously noted for both SHa [29] and SHf [73].

Analysis of the leishmanicidal mechanism revealed that SHa and [K^3^]SHa act very rapidly on promastigotes, within 5–15 min. Similar to bacteria, we observed distinct mechanisms of action on parasites based on the peptide concentration. At a concentration of 10 *μ*M, the killing activity of SHa and [K^3^]SHa was correlated with the permeabilization/depolarization of the plasma membrane, without the release of macromolecular complexes (luciferase leakage) and loss of plasma membrane integrity (PI staining). In contrast, at concentrations above 10 *μ*M, both temporins induced a luciferase leakage and the loss of cell membrane integrity in a dose-dependent manner. Membrane alterations (blebbing) were clearly evidenced by AFM and FEG-SEM imaging following treatment with temporins. To our knowledge, this is the first AFM study showing the effect of temporins on *Leishmania* and *Trypanosoma* parasites. Membrane blebbing is also a hallmark of apoptotic cell death [74], which has been observed during *Leishmania* apoptotic cell death following chronic heat exposure [75]. Therefore, these results do not exclude the possibility that other mechanisms may also mediate temporin-induced cell death. Kulkarni and coworkers showed that AMPs with different structures can kill *Leishmania* by distinct mechanisms. For example, magainin-type AMPs induced notable changes (changes in surface permeability, DNA degradation, depolarization of the mitochondrial membrane potential, caspase-3/7-like activation) consistent with apoptosis, while the cathelicidin-type AMPs kill *Leishmania* by a non-apoptotic mechanism [76]. Hence, we investigated whether SHa and [K^3^]SHa could induce apoptotic-like death. Cellular events, such as mitochondrial membrane depolarization (a marker of early apoptosis) or DNA fragmentation (a marker of late apoptosis), were observed after exposure of *Leishmania* promastigotes to temporins. This suggests that, in addition to their primary membranolytic effect, temporins may trigger other cell death mechanisms at concentrations above the IC_50_.

From our studies, [K^3^]SHa has emerged as a promising analog, with potent broad-spectrum activity (Gram-negative and Gram-positive bacteria, yeasts and parasites), low resistance susceptibility and the ability to efficiently kill intracellular forms of *Leishmania*. As attractive small lead compounds, SHa and its analogs open the way to the design of a new class of peptide-based drugs effective against resistant bacteria and parasites. Although delineating the *in vivo* toxicity and immunological activity of SHa and [K3]SHa is needed to further investigate their *in vivo* antibacterial and antiparasitic therapeutic potential, SHa and its analogs represent attractive small lead compounds that open the way to the design of a new class of peptide-based drugs effective against resistant bacteria and parasites.

## Materials and methods

### Peptide synthesis

SHa and its analogs, [K^3^]SHa, [A^2,6,9^]SHa, [A^2,6,9^, [A^2,6,9^, K^3^]SHa and D-temporin-SHa (D-SHa), were synthesized using solid-phase standard FastMoc chemistry protocols on an Applied Biosystems 433A automated peptide synthesizer (Peptide Synthesis Platform, IBPS, FR 3631 UPMC-CNRS, UPMC, Paris, France), as previously described [77]. The homogeneity and identity of the synthetic peptides were assessed by analytical reversed-phase high-performance liquid chromatography (RP-HPLC) on a C18 analytical column (Modulo-cart QS Uptisphere 5 ODB, 5 mm, 250 x 4.6 mm, Interchim, Montluçon, France) and by matrix-assisted laser desorption/ionization time-of-flight (MALDI-TOF) mass spectrometry carried out on a Voyager-DE PRO (Applied Biosystems, Foster City, CA, USA) under the same conditions as described previously (Mass Spectrometry and Proteomics Platform, IBPS, FR 3631 UPMC-CNRS, UPMC, Paris, France) [77].

### Microorganisms, cell lines and growth conditions

Several strains, including bacteria, yeasts/fungi and parasites, were used in our study to assess antimicrobial activity.

#### Bacteria

*Staphylococcus aureus* ATCC 25923 (ATCC: American Type Culture Collection), *S*. *aureus* ST1065 (kindly provided by Tarek Msadek, Institut Pasteur Paris, France), multidrug-resistant *S*. *aureus* ATCC 43300 and ATCC BAA-44 (ATCC), *Enterococcus faecalis* ATCC 29212 (ATCC), *Bacillus megaterium* (laboratory collection), *Escherichia coli* ATCC 25922, *E*. *coli* ML-35p, *Salmonella enterica* serotype Enteritidis (kindly provided by Sylvie Rebuffat, Muséum National d'Histoire Naturelle, Paris, France), *Pseudomonas aeruginosa* ATCC 27853, *Klebsiella pneumoniae* ATCC 13883, and *Acinetobacter baumannii* ATCC 19606 (ATCC) were incubated in lysogeny broth (LB, Sigma-Aldrich) for 2–3 h at 37°C to obtain a mid-logarithmic phase culture. *Listeria ivanovii* Li4pVS2 (kindly provided by Jean-Marc Berjeaud, Université de Poitiers, France), and *Streptococcus pyogenes* ATCC 19615 (ATCC) were cultured in brain heart infusion (BHI, Sigma-Aldrich) medium under the same conditions.

#### Yeasts/Fungi

*Candida albicans* ATCC 90028, *Candida parapsilosis* ATCC 22019 (ATCC), and *Saccharomyces cerevisiae* (laboratory collection) were cultured in yeast peptone dextrose (YPD, Sigma-Aldrich) medium at 30°C.

#### Parasites

*Leishmania infantum* (strain MHOM/MA/67/ITMAP-263), *Leishmania major* (strain MHOM/SU/73/5-ASKH), *Leishmania tropica* (strain MHOM/SU/74/SAFK27), *Leishmania amazonensis* (strain MHOM/BR/73/M2269), and *Leishmania braziliensis* (strain MHOM/BR/75/M2904) promastigotes (kindly provided by Francine Pratlong, Centre National de Référence des Leishmania, Montpellier, France) were cultured at 26°C in SDM-79 medium supplemented with 10% fetal calf serum (FCS, Gibco) [23]. *L*. *infantum* axenic amastigotes were differentiated from promastigotes in MAA medium (supplemented with 20% FCS) and were grown at 37°C in 5% CO_2_ [23]. *L*. *infantum*-infected human macrophages were obtained from human THP-1 monocytes, as previously described [23, 78]. The procyclic form of *Trypanosoma brucei gambiense* (strain MHOM/CI/86/DAL967) and the epimastigote form of *Trypanosoma cruzi* (strain Y cl7 scl2, lineage Tc2) (kindly provided by Philippe Truc and Christian Barnabé, UMR 177 IRD-CIRAD InterTryp, Montpellier, France) were grown at 260078C in Cunningham’s medium and liver infusion tryptose (LIT) medium, respectively, supplemented with 10% FCS, 2 mM GlutaMAX™-I, 100 U/ml penicillin, 100 *μ*g/ml streptomycin and bovine hemin (20 mg/l) (Gibco) [23].

#### Human cell lines

THP-1 monocytes (kindly provided by Ali Ouaissi, Institut Pasteur Lille, France) were cultivated in RPMI medium supplemented with 10% FCS, 2 mM GlutaMAX™-I, 100 U/ml penicillin, and 100 *μ*g/ml streptomycin. Hepatocarcinoma-derived cells (HepG2, kindly provided by Martine Daujat, Hôpital Saint Eloi, Montpellier, France) were adapted in minimum essential medium (MEM) with Earle’s salts supplemented with 10% FCS, 10 mM HEPES, 1 mM sodium pyruvate, 2 mM GlutaMAX™-I, 100 UI/ml penicillin, 100 *μ*g/ml streptomycin, and MEM non-essentials amino acids (100 *μ*M each). Human foreskin fibroblasts (HFF ATCC SCRC-1042) were kindly provided by Corinne Loeuillet (Université Grenoble-Alpes, France) and were cultivated in Dulbecco's modified Eagle’s medium (DMEM) supplemented with 10% FCS, 2 mM GlutaMAX™-I, 100 U/ml penicillin, and 100 *μ*g/ml streptomycin. THP-1, HepG2 and HFF cells were maintained in a 5% CO_2_ incubator at 37°C.

### Antimicrobial assays

Activity against bacteria and yeasts was assessed with a liquid growth inhibition assay performed in 96-well microtitration plates, according to a previously described protocol [14], using mid-logarithmic phase cultures diluted in Mueller-Hinton (MH, Dominique Dutscher) broth (LB broth for *E*. *faecalis* strain and YPD medium for yeasts/fungi) to an A_630_ value of 0.01 (10^6^ cfu/ml) and 2-fold serial dilutions of synthetic temporin (200–1 *μ*M, final concentrations). The minimal inhibitory concentration (MIC) was determined as the mean value of three independent experiments, each performed in triplicate with negative (H_2_O) and positive (0.7% formaldehyde) controls. Antiparasitic activity was evaluated against trypanosomatids, such as *Leishmania* and *Trypanosoma*. Antileishmanial activity was determined either by a luminescence-based assay (*L*. *infantum*, *L*. *major* and *L*. *braziliensis*) or by cell counting (FACSCalibur flow cytometer, Becton-Dickinson Biosciences, Woburn, MA, USA) after staining with propidium iodide (*L*. *tropica and L*. *amazonensis*), as previously described [23], using promastigotes or axenic amastigotes (1.25 x 10^6^ parasites/ml) in mid-log phase and 5-fold serial dilutions of synthetic temporin (50–3.1 *μ*M, final concentrations). Antitrypanosomal activity against *T*. *brucei gambiense* and *T*. *cruzi* was assessed by cell counting, similar to the *L*. *tropica and L*. *amazonensis* analysis, after incubation of the parasites (96 h for *T*. *brucei gambiense* and 120 h for *T*. *cruzi*) with the synthetic temporin, as previously described [23]. The half maximal inhibitory concentration (IC_50_) value was determined with GraphPad Prism 6.0 software (GraphPad Software, La Jolla, CA, USA) using a sigmoidal dose response (variable slope) curve fitting equation. The results are expressed as the means of three independent experiments performed in triplicate (luminescence-based assays) or in duplicate (cell counting-based assays).

### Multipassage resistance selection

To assess the possibility of bacterial resistance selection under temporin pressure, multipassage resistance selection studies were performed with SHa, D-SHa, a more stable analog corresponding to SHa with all residues in D-configuration, and [K^3^]SHa using *E*. *coli* ATCC 25922. Ampicillin was also tested in the assay as a conventional antibiotic, and sterile MilliQ H_2_O was used as the negative control. *E*. *coli* bacteria were cultured in MH broth for 24 h at 37°C with shaking at 200 rpm. Ten microliters of the bacterial suspension were re-inoculated once a day in 990 *μ*l of MH media for five days. Following re-inoculation, the remaining bacterial suspension (990 *μ*l) was kept frozen (–80°C) each day. After these five initial passages in unsupplemented MH media, *E*. *coli* was then continuously re-inoculated (10 *μ*l) once a day in 990 *μ*l of MH media containing a constant concentration of antimicrobial agent (temporin or ampicillin) for ten days (10 passages at 1/16 MIC as a starting concentration). This procedure was repeated with a 2-fold higher concentration of antimicrobial agent for additional ten days (i.e. 10 passages at 1/8 MIC) and then the concentration was doubled until reaching the MIC (i.e. 10 passages at 1/4 MIC, 1/2 MIC, and MIC). E. coli lineages resulting from these passages in supplemented MH media (antimicrobial agent) were daily kept frozen at –80°C, like for the five initial passages in unsupplemented MH media. Finally, the MIC of the antimicrobial agent was determined against the adapted *E*. *coli* lineages originating from different last passages (passage 5: *E*. *coli* with no antimicrobial agent; 15: *E*. *coli* with a concentration of antimicrobial agent equal to 1/16 MIC; 25: 1/8 MIC; 35: 1/4 MIC; 45: 1/2 MIC; 55: MIC) by using the same protocol described above for bacteria (Antimicrobial Assays). MIC values were obtained in triplicate and represent the average of three independent experiments. Curves representing the MIC as a function of the passage number were obtained from the means ± SEM of MIC values of at least three independent experiments.

### Time-kill assays

Time-kill assays were performed on bacteria and *Leishmania infantum* promastigotes. For bacteria (*E*. *coli* ML-35p and *S*. *aureus* ST1065, 10^6^ cfu/ml), assays were performed as previously described [23], with final temporin concentrations of 6 and 12 *μ*M. For parasite time-kill assays, *Leishmania infantum* promastigotes harvested in exponential phase were washed twice (1,000 x g, 10 min) with Hank's balanced salt solution (HBSS) supplemented with 20 mM glucose and then suspended in the same buffer to a density of 2.5 x 10^6^ cells/ml. This suspension (800 *μ*l) was incubated with 200 *μ*l of a 5X temporin solution, and parasitic death was evaluated at different times by counting parasites (FACSCalibur) after staining with 1 *μ*g/ml propidium iodide (Molecular Probes). Negative controls were assayed without peptides. Three experiments were carried out in triplicate for bacteria or in duplicate for parasites.

### Intramacrophagic killing of *Leishmania*

Intramacrophagic leishmanicidal activity of the peptides was assessed by incubating *L*. *infantum*-infected human macrophages with 2-fold serial dilutions of peptide (50–2.5 *μ*M, final concentrations) for 4 days, as previously described [23]. THP-1-derived macrophages were infected with promastigotes from late log phase at a density of 5 x 10^5^ cells/well (ratio 10:1) in RPMI medium for 24 h at 37°C with 5% CO2. In contrast to IC_50_ values were determined with GraphPad Prism 6.0 software. The results are expressed as the means of two experiments performed with six replicates.

### Permeabilization assays

The ability of SHa and its analogs to permeate the bacterial cytoplasmic membrane was determined by two methods using either the β-galactosidase substrate *o*-nitrophenyl-β-D-galactopyranoside (ONPG, Sigma-Aldrich) or the high-affinity nucleic acid dye SYTOX Green (SG, Life Technologies). For the first assay, peptide-induced permeabilization of *E*. *coli* (strain ML-35p) was measured by the rate of production of *o*-nitrophenol (ONP) at 405 nm following hydrolysis of ONPG by cytoplasmic β-galactosidase, as previously described [14]. We also analyzed the release of cytoplasmic β-galactosidase in the extracellular medium upon temporin treatment, according to a previous protocol [14], and used melittin and dermaseptin B2 as positive controls. Two experiments were carried out in triplicate. The results are expressed as the means ± SEM after subtraction of the negative control values (no peptide) from the test values. For the SG uptake assay, we used *Klebsiella pneumoniae* and *Streptococcus pyogenes* as Gram-negative and Gram-positive species, respectively. Fifty microliters of bacteria (10^6^ cfu/ml) resuspended in PBS containing 1 *μ*M of SG was preincubated in 96-well microtitration plates for 20 min at 37°C in the dark. After addition of peptide (50 *μ*l) at different concentrations, the fluorescence was measured every 5 min for 60 min at 37°C using 485 and 520 nm filters for excitation and emission wavelengths, respectively (FLUOstar Galaxy spectrofluorometer, BMG Labtech, Champigny-sur-Marne, France). SG cannot enter live cells, but it penetrates the cell and binds to intracellular DNA when the cell membrane is damaged, leading to an intense green fluorescence.

To evaluate peptide-induced alterations of the parasite membrane, fluorometric measurements of SG influx were first performed on *L*. *infantum* promastigotes and *T*. *cruzi* epimastigotes. The protocol was similar to that used for bacteria. Eighty microliters of parasites (2.5 x 10^6^ cells/ml) in HBSS supplemented with 20 mM glucose was incubated in 96-well tissue culture microplates 1 *μ*M of SG for 20 min in the dark at 26°C. After the addition of 20 *μ*l peptide at different concentrations, the increase in fluorescence was measured (Wallac 1420 VICTOR2 multilabel microplate reader, Perkin Elmer, Waltham, MA, USA) under the same conditions described above. Maximal membrane permeabilization was defined as that obtained after the addition of 0.1% Triton X-100, Sigma-Aldrich). Propidium iodide (PI) was also used as a marker of membrane integrity of parasites. Eight hundred microliters of *L*. *infantum* promastigotes (1.25 x 10^6^ cells/ml in SDM-79 medium) was incubated with 200 *μ*l of peptide at different concentrations (10, 20 and 40 *μ*M, final concentrations). Aliquots of 40 *μ*l were taken at different times and diluted in 460 *μ*l of PBS containing PI (1 *μ*g/ml). The PI signal, which is correlated with mortality, was then assessed by a flow cytometer (FACSCalibur) equipped with an argon ion laser set at 488 nm excitation and a long-pass emission filter of 650 nm for red fluorescence. The percentage of PI-positive cells was calculated as follows = [% of PI positive cells (parasites incubated with peptide)–% of PI positive cells (parasites incubated with PBS)]. To assess membrane permeabilization of the parasites, we employed a third complementary assay that consists of incubating 800 *μ*l of a suspension of *L*. *infantum* promastigotes expressing luciferase activity (10^6^ cells/ml) in SDM-79 medium with 200 *μ*l of peptide at different concentrations (10, 20 and 40 *μ*M, final concentrations). Aliquots of 100 *μ*l were taken at different times and centrifuged (5,000 x g, 5 min), and 40 *μ*l of the supernatant was added to a tissue culture microplate. After reaction with 20 *μ*l of Steady-Glo buffer (Steady-Glo Luciferase Assay System, Promega), the luminescence was measured (Wallac 1420 VICTOR2 microplate reader). The positive control was assayed by measuring luciferase activity of promastigotes and the negative control by measuring the luciferase activity in the supernatant of a parasite culture without peptides. The data are expressed as the means ± SEM of two experiments carried out in triplicate after subtraction of the negative control values (no peptide) from the test values.

### Membrane depolarization assays

Peptide-induced depolarization of both bacterial and parasite plasma membranes was analyzed using the membrane potential-sensitive fluorescence probe DiSC_3_(5) (3,3′-dipropylthiadicarbocyanine iodide, Molecular Probes). Membrane depolarization of *S*. *aureus* ATCC 25923 was assessed using a previously described protocol [73]. *Leishmania* promastigotes (*L*. *infantum* and *L*. *amazonensis*) and *T*. *cruzi* epimastigotes were used to determine whether temporins could perturb the potential gradient of the parasite membrane. Parasites at exponential phase were washed twice with HBSS supplemented with 20 mM glucose and 5.7 mM KCl and then diluted to a density of 1.25 x 10^7^ cells/ml. Then, 792 *μ*l of the parasite suspension was transferred to a fluorescence cuvette, and 8 *μ*l of 250 *μ*M DiSC_3_(5) was added. Once the probe was completely captured by the cells, 200 *μ*l of the peptide (SHa or [K^3^]SHa) was added at a final concentration of 50 *μ*M, and fluorescence was monitored at 26°C for 20 min at the same excitation and emission wavelengths used for bacteria experiments. Three independent experiments were performed with no peptide as the negative control.

### Cytotoxic activities

The cytotoxic activities of SHa and its analog, [K^3^]SHa, were evaluated using human erythrocytes and human cell lines, including THP-1 monocytes, THP-1-derived macrophages, HFF fibroblasts and HepG2 cells. Hemolytic assays were also performed using erythrocytes obtained from fresh blood samples of Wistar male rats (Charles River Laboratories, France). The hemolytic activity was measured by incubating the peptide (1–200 *μ*M, final concentrations) with erythrocytes (2 x 10^7^ cells) in PBS (100 *μ*l) for 1 h at 37°C, as previously described [23]. A parallel incubation in the presence of 0.1% (v/v) Triton-X100 was carried out to determine the absorbance associated with 100% hemolysis. Viability of the THP-1, HepG2, and HFF cells was determined with 3-(4,5-dimethylthiazolyl-2)-2,5-diphenyltetrazolium bromide (MTT) after incubation of the cells (THP-1 and HepG2: 5 x 10^5^ cells/ml; HFF: 10^5^ cells/ml) for 72 h at 37°C with peptide (THP-1: 1–60 *μ*M; HepG2: 50–600 *μ*M; HFF: 1–100 *μ*M; final concentrations), as previously detailed [77]. For THP-1-derived macrophages, viability was determined by Trypan blue staining after incubation with peptide (1–100 *μ*M, final concentrations). LC_50_ (average concentration of the peptide producing 50% cell lysis) and IC_50_ values were determined with GraphPad Prism 6.0 software and correspond to the mean obtained from three independent experiments carried out with three (erythrocytes, THP-1 monocytes, HepG2 and HFF cells) or six replicates (THP-1-derived macrophages).

### Therapeutic index calculation

The therapeutic index was calculated according to Chen and collaborators [79] as the ratio of IC_50_ or LC_50_ values over the MIC_GM_ values against several strains. IC_50_ or LC_50_ values were determined for the different human cells mentioned above in the cytotoxicity assays. For values higher than the maximum concentration tested (serial 2-fold dilutions), a minimal 2-fold concentration value was used to calculate the therapeutic index (i.e. LC_50_ > 100 was considered as 200). MIC_GM_ correspond to the geometric mean (GM) of peptide MIC values determined against several strains: 6 Gram-negative bacteria, 7 Gram-positive bacteria and 3 yeasts/fungal strains.

### Measurement of mitochondrial membrane potential (Δψ_m_)

*Leishmania* mitochondrial membrane potential was assessed using the cationic and cell-permeable fluorescent probe tetramethylrhodamine ethyl ester (TMRE, Molecular Probes). *L*. *infantum* promastigotes were harvested in the exponential phase, washed twice with HBSS (supplemented with 20 mM glucose), and adjusted to a density of 2.5 x 10^6^ cells/ml. Then, 1.6 ml of the cell suspension was incubated for 20 min with 100 ng/ml of TMRE in the dark at 26°C. Next, 400 *μ*l of two concentrations of peptide (3 and 6 *μ*M, final concentrations) were added, and changes in the mitochondrial membrane potential were monitored at different times (0, 5, 15, 30, 60, 120 and 180 min) by flow cytometry (λ_ex/em_ = 549/574 nm, FACSCalibur). Negative and positive controls were assayed without peptide or with 0.5 mM carbonyl cyanide m-chlorophenylhydrazone (CCCP, Sigma-Aldrich), respectively. Alterations in TMRE fluorescence were quantified using an index of variation (IV), which was calculated using the following formula: IV = (MFI_t_−MFI_c_)/MFI_c_, where MFI_t_ is the median fluorescence for treated parasites and MFI_c_, that of control parasites [80]. Negative IV values correspond to depolarization of the mitochondrial membrane. Three independent experiments were performed.

### Apoptosis detection by cell cycle analysis and TUNEL (terminal deoxynucleotidyl transferase-mediated dUTP nick-end labeling) assay

Mid-log phase *L*. *infantum* promastigotes were synchronized at the G1/S border by treating with 10 mM hydroxyurea for 24 h at 26°C [81]. Parasites were harvested, washed twice with SDM-79 medium (1,000 x g, 10 min) and adjusted to a density of 1.25 x 10^6^ cells/ml. Then 400 *μ*l of different concentrations of peptide (12.5, 25 and 50 *μ*M, final concentrations) were added to 1.6 ml of the cell suspension. After 48 h incubation, samples were split into two volumes (2 x 1 ml) to analyze the cell cycle and DNA fragmentation. For cell cycle analysis, the first sample (1 ml) was centrifuged (1,000 x g, 10 min) and 100 *μ*l of HBSS was added to the pellet. Then, 900 *μ*l of cold 70% ethanol was gently added drop-wise for cell fixation. After an overnight incubation at -20°C, cells were washed (16,000 x g, 10 min, 4°C) with HBSS and resuspended in 1 ml of HBSS containing 100 μg of RNase A (Sigma-Aldrich) and 5 μg of propidium iodide. After 30 min of incubation at 37°C, samples were passed 5 times through a sterile plastic syringe fitted with a 27 gauge needle and analyzed by flow cytometry (FACSCalibur). For DNA fragmentation analysis, a TUNEL assay was performed on the second 1 ml sample. The sample was centrifuged (1,000 x g, 10 min), and the pellet was fixed with 2% formaldehyde on ice for 15 min. Cells were centrifuged again (16,000 x g, 10 min, 4°C) and 1 ml of cold 70% ethanol was gently added drop-wise. After overnight incubation at -20°C, the pellet was subjected to the TUNEL labeling reaction using the FlowTACS™ apoptosis detection kit (Trevigen, Gaithersburg, MD, USA) according to the manufacturer’s protocol. DNA strand breaks were detected by the addition of biotinylated nucleotides to the free 3′-hydroxyl ends of the DNA fragments using TdT (terminal deoxynucleotidyl transferase) and tagged with a streptavidin-FITC conjugate for subsequent detection by flow cytometry. For both experiments, negative and positive controls were assayed without peptide or with 50 μM miltefosine (Sigma-Aldrich), respectively. The data were processed using FlowJo vX.0.7 software, and the results are shown as the means ± SEM of three independent experiments.

### Circular dichroism (CD)

The CD spectra of temporins (30 μM) were recorded with a Jobin Yvon CD6 spectropolarimeter at 25°C in PBS (10 mM phosphate, pH 7.3) containing 1 mg/ml DMPC/DMPG 3:1 (mol:mol) large unilamellar vesicles (LUVs), as previously described [14]. DMPC, dimyristoyl phosphatidylcholine; DMPG, dimyristoyl phosphatidylglycerol.

### Nuclear magnetic resonance (NMR) spectroscopy

Samples were prepared in a volume of 300 μl in Shigemi tubes and contained 1 mM peptide, 50 mM dihexanoyl phosphatidylcholine (DHPC) and 25 mM DMPG (Avanti Polar Lipids, Alabaster, AL, USA) in 50 mM sodium phosphate, pH 6.4, 10% D_2_O, 0.02% (w/v) NaN_3_ and 0.1 mM sodium 2,2-dimethyl-2-silapentane- 5-sulfonate-*d*_6_ (Sigma Aldrich, St. Louis, MO, USA). NMR experiments were recorded on a Bruker Avance III 500 MHz spectrometer equipped with a 5 mm TCI cryoprobe. ^1^H resonances were assigned using 2D TOCSY (DIPSI-2 isotropic sequence of 40 and 60 ms) and 2D NOESY spectra (75 and 150 ms mixing times) recorded at 37°C. Experiments were acquired using a double pulsed field gradient spin echo with band-selective pulses (90° read pulse of 4 ms duration and G4 shape and 180° REBURP pulses of 3 ms duration) centered on the amide/aromatic region. For the paramagnetic experiments, 1-palmitoyl-2-stearoyl-(12-doxyl)-*sn*-glycero-3-phosphocholine (12-doxylPC, Avanti Polar Lipids) was added at a concentration of 0.5 mM.

### Surface plasmon resonance (SPR)

Real-time and free label peptide-liposome interaction assays were performed on the Molecular Interactions Platform (FR 3631 UPMC-CNRS, IBPS, Paris, France) using sensor chip L1 and a Biacore 3000 instrument (GE Healthcare, Uppsala, Sweden) at 25°C. Liposomes corresponding to DMPC/DMPG 3:1 (mol:mol) LUVs were prepared according to a previous protocol (15). Multilamellar vesicles (MLVs, 1 mg/ml) were obtained by hydrating the dry lipid film with HBS-N (10 mM HEPES pH 7.4, 150 mM NaCl; GE Healthcare, Uppsala, Sweden). After several rounds of freeze-thawing, the lipid sample was extruded with 10 successive passages through polycarbonate membranes of reduced pore size (400, 200 and 100 nm), using an extruder (Avanti Polar Lipids) preheated at 50°C for 10 min. DMPC/DMPG LUVs (1 mg/ml) were kept at room temperature and used for SPR experiments within 24 h. HBS-N was used as the running buffer. Prior to immobilization of the LUVs, the L1 sensor chip was conditioned by the injection of 40 mM *n*-octyl-β-D-glucopyranoside (OG, Sigma-Aldrich) (flow rate, 5 μl/min; contact time, 60 s), and bovine serum albumin was injected (0.2 mg/ml; flow rate, 5 μl/min; contact time, 600 s) to prevent non-specific interactions of the peptide. Then, DMPC/DMPG liposomes (0.2 mg/ml) were injected (flow rate, 1 μl/min; contact time, 120 s). Peptides in HBS-N running buffer were injected on the immobilized surface at a flow rate of 20 μl/min with a contact time of 60 s. Dissociation kinetics were then assayed for 600 s. The sensor chip surface was completely regenerated using 40 mM OG as the regeneration buffer (flow rate, 5 μl/min; contact time, 60 s). Kinetic studies were performed with different concentrations of SHa and [K^3^]SHa (100, 150, 200, 250 and 300 nM), and the data were plotted using BIAevaluation software 4.1. The affinity, corresponding to the equilibrium dissociation constant K_D_, was determined by the kinetic simultaneous k_a_/k_d_ model, assuming Langmuir (1:1) binding.

### Differential scanning calorimetry (DSC)

The interaction of temporins with membrane models was assessed by DSC, as previously described [14]. Different peptide:lipid molar ratios (1:100 and 1:50) were assessed by mixing the appropriate volumes of the MLV suspension (DMPG or DMPC) and peptide solution (1 mM). Thermodynamic values (T_m_ and ΔH) were estimated by a peak-fitting procedure using CpCalc software.

### Atomic force microscopy (AFM) imaging of bacteria (*P*. *aeruginosa*) and parasites (*L*. *infantum* and *T*. *cruzi*)

*P*. *aeruginosa* bacteria (4 x 10^7^ cells/ml) were incubated for 1 h with temporins (SHa, 50 μM; [K^3^]SHa, 6 and 12 μM; final concentrations) in LB medium at 37°C. After washing with PBS, the cells were fixed 1 h with 2.5% glutaraldehyde/PBS. Parasites (*L*. *infantum* promastigotes or *T*. *cruzi* epimastigotes, 1 x 10^6^ cells/ml) were incubated for 30 min with [K^3^]SHa (5 *μ*M, final concentration) in RPMI medium at 26°C. After centrifugation, the cells were fixed with 2% formaldehyde. The different samples containing bacteria or parasites were then deposited on glass surfaces and dried with ultrapure N_2_. AFM images of the dried surfaces were recorded as described [82] using a commercial di Caliber AFM microscope (Bruker Instruments Inc., Camarillo, CA, USA). To avoid tip and sample damage, topographic images were taken in the non-contact dynamic mode (tapping® mode). Silicon nitride tips (resonance frequency of 280–400 kHz, force constant of 40–80 N/m) were used. Images were obtained at a constant speed of 1 Hz with a resolution of 512 lines of 512 pixels each. The raw data were processed using Nanoscope Analysis v.1.30 imaging processing software (Bruker Instruments Inc., Camarillo, CA, USA) and were used mainly for sample slope correction and 3D image processing. AFM analyses were carried out with at least at three different locations on each surface, with a minimum of 100 single bacteria/parasites observed.

### Field emission gun-scanning electron microscopy (FEG-SEM) imaging of *L*. *infantum* promastigotes

FEG-SEM images were recorded as described previously [82] with a Hitachi SU-70 Field Emission Gun Scanning Electron Microscope. The samples were fixed on an alumina SEM support with carbon adhesive tape and were observed without metallization. A scintillator secondary electron detector [SE-Lower, SE(L)] was used to characterize the dried inoculums deposited on gold surfaces. The accelerating voltage was 1 kV, and the working distance was approximately 16.2 mm. At least five different locations were analyzed on each surface, observing a minimum of 100 single parasites.

## Supporting information

S1 Fig **Effect of [K**^**3**^**]SHa on the thermotropic phase behavior of DMPG (A) and DMPC (B) MLVs compared to SHa.** Variations of the main phase transition temperature (T_m_) and total enthalpy (ΔH) values for gel-to-liquid crystalline transition of MLVs are indicated as ΔT_m_ (T_m_—T_m w/o peptide_) and % Δ(ΔH) [(ΔH - ΔH _w/o peptide_) x 100 / ΔH _w/o peptide_]. T_m_ and ΔH values were estimated by a peak-fitting procedure using CpCalc software and correspond to the mean ± SEM obtained from six scans.
(TIFF)

S1 Movie Leishmanicidal activity of temporin-SHa. (MOV)

S2 Movie Trypanocidal activity of temporin-SHa. (MOV)

S1 Table ^1^H NMR chemical shifts of temporin-SHa in DHPC/DMPG bicelles (500 MHz, 37°C). (DOCX)

S2 Table ^1^H NMR chemical shifts of [K^3^]temporin-SHa in DHPC/DMPG bicelles (500 MHz, 37°C). (DOCX)
