# Supplementary figures and images for: Insight into the mechanism of action of temporin-SHa, a new broad-spectrum antiparasitic and antibacterial agent
Source: PLoS One. 2017 Mar 20;12(3):e0174024. doi: 10.1371/journal.pone.0174024 (PMC5358776; doi:10.1371/journal.pone.0174024)

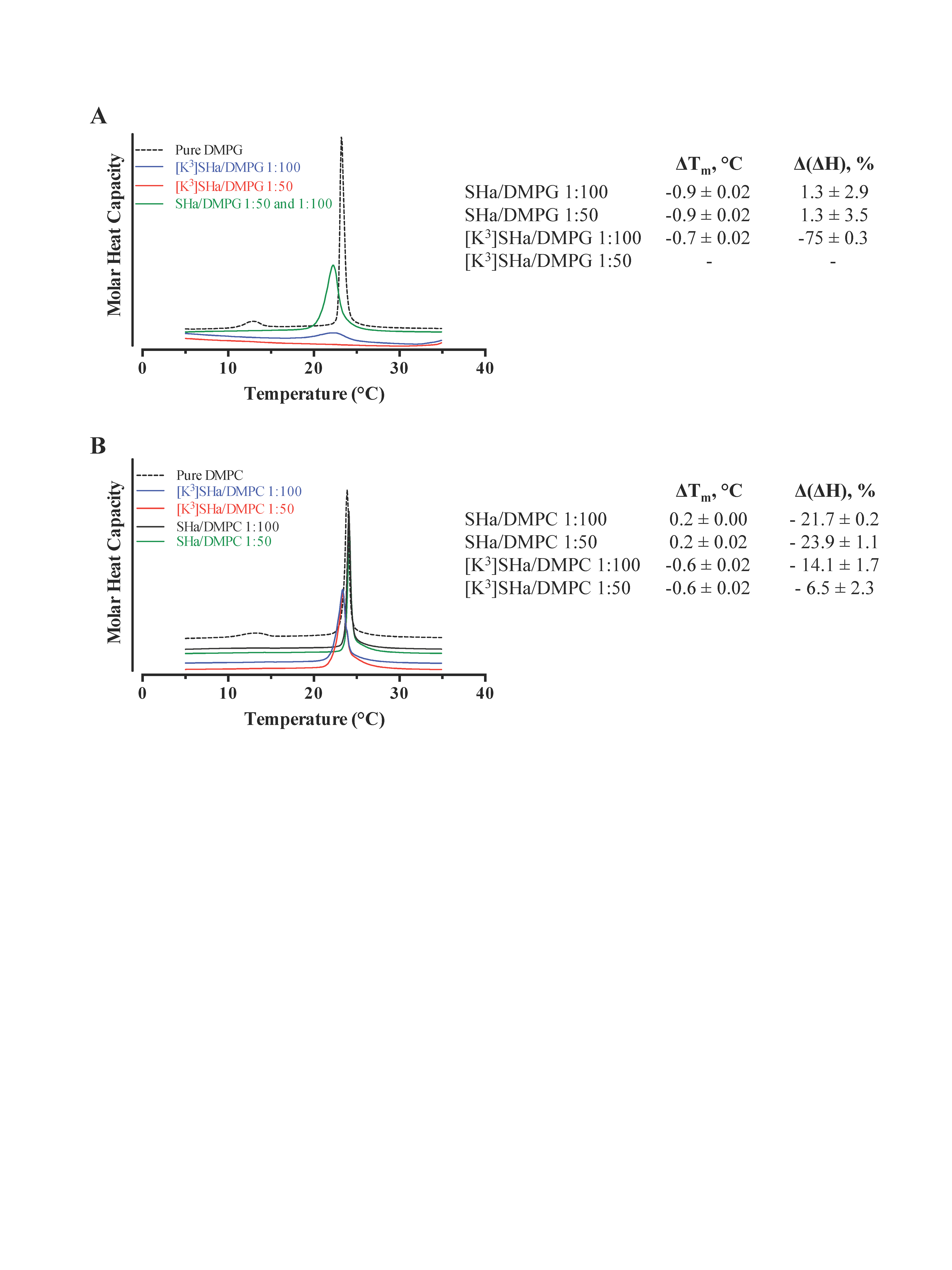

Supplement: S1 Fig — Effect of [K3]SHa on the thermotropic phase behavior of DMPG (A) and DMPC (B) MLVs compared to SHa. Variations of the main phase transition temperature (Tm) and total enthalpy (ΔH) values for gel-to-liquid crystalline transition of MLVs are indicated as ΔTm (Tm—Tm w/o peptide) and % Δ(ΔH) [(ΔH - ΔH w/o peptide) x 100 / ΔH w/o peptide]. Tm and ΔH values were estimated by a peak-fitting procedure using CpCalc software and correspond to the mean ± SEM obtained from six scans. (TIFF) [file pone.0174024.s001.tiff]
