# Supplementary material for: Insight into the mechanism of action of temporin-SHa, a new broad-spectrum antiparasitic and antibacterial agent
Source: PLoS One. 2017 Mar 20;12(3):e0174024. doi: 10.1371/journal.pone.0174024 (PMC5358776; doi:10.1371/journal.pone.0174024)
Supplement: S1 Table — (DOCX) [file pone.0174024.s004.docx]

| **Residue** | **HN** | **Hα** | **Hβ** | **Other side chain protons** |
| --- | --- | --- | --- | --- |
| Phe^1^ |  | 4.20 | 3.31, 3.21 | Hδ 7.32; Hε 7.28; Hζ 7.17 |
| Leu^2^ | n.d. | 4.03 | 1.67 | Hδ 0.86 |
| Ser^3^ | 8.65 | 4.15 | 3.94 |  |
| Gly^4^ | 8.48 | 4.00 |  |  |
| Ile^5^ | 7.86 | 3.95 | 2.02 | Hγ1 1.62, 1.22; Hγ2 0.93; Hδ1 0.85 |
| Val^6^ | 8.26 | 3.58 | 2.19 | Hγ 1.07, 0.99 |
| Gly^7^ | 8.22 | 3.94 |  |  |
| Met^8^ | 7.85 | 4.24 | 2.27, 2.14 | Hγ 2.68, 2.55; Hε 2.05 |
| Leu^9^ | 8.26 | 4.10 | 2.05, 1.51 | Hγ 2.05; Hδ 0.91 |
| Gly^10^ | 8.38 | 3.98, 3.92 |  |  |
| Lys^11^ | 7.59 | 4.18 | 1.95 | Hγ 1.60, 1.52; Hδ 1.71 |
| Leu^12^ | 7.69 | 4.04 | 1.54, 1.13 | Hγ 1.5; Hδ 0.81, 0.72 |
| Phe^13^ | 7.76 | 4.60 | 3.35, 2.90 | Hδ 7.39 Hε 7.17; Hζ 7.05 |

^a^Chemical shifts were referenced to internal DSS (sodium 2,2-dimethyl-2-silapentane-*d*_6_-5-sulfonate).
